# Acclimation to white light in a far‐red light specialist: insights from *Acaryochloris marina* MBIC11017

**DOI:** 10.1111/nph.70188

**Published:** 2025-05-05

**Authors:** Thomas J. Oliver, Eduard Elias, Roberta Croce

**Affiliations:** ^1^ Department of Physics and Astronomy, Faculty of Sciences, Institute for Lasers, Life and Biophotonics Vrije Universiteit Amsterdam de Boelelaan 1100 Amsterdam HZ, 1081 the Netherlands

**Keywords:** *Acaryochloris*, chromatic acclimation, far‐red light, light harvesting, photosynthesis, photosystems

## Abstract

- The Chl *d*‐containing cyanobacterium, *Acaryochloris marina* MBIC11017, is constitutively adapted to far‐red light (FRL). However, it occasionally encounters white light (WL) in its natural habitat. Using biochemical and spectroscopic techniques, we investigated how this organism acclimates to WL and analysed the excitation energy trapping dynamics of its photosystems and complex antenna system, comprised of both membrane‐embedded and soluble antenna.
- When grown in WL, *A. marina* MBIC11017 doubles its Photosystem I/Photosystem II (PSI/PSII) ratio and increases its phycobilisome content compared with FRL, without altering their composition, while the number of membrane‐embedded antennae decreases. Under both light conditions, phycobilisomes primarily transfer excitation energy to PSII, but a smaller fraction transfers to PSI.
- The PSI trapping time is fast (35 ps), confirming the absence of red‐shifted forms. By contrast, PSII trapping is slower, with two components of *c.* 115 and *c.* 480 ps. Simulations based on the PSII structure suggest that this slow trapping arises mainly from the PSII antenna arrangement rather than from the use of Chl*d* as a primary donor.
- These results reveal how *A. marina* MBIC11017 dynamically adjusts photosystem ratios and antenna composition to changes in light quality, offering insights into the ecological and functional implications of Chl*d*‐driven photosynthesis and chromatic acclimation.

## Introduction

Oxygenic photosynthesis converts solar energy into chemical energy using two protein–pigment complexes, Photosystem I (PSI) and Photosystem II (PSII). In most oxygenic phototrophs, these photosystems use Chl*a* as a light‐harvesting pigment and a redox‐active molecule, absorbing light and performing charge separation, so the photosystems can perform their catalytic functions. For many years, it was thought that this process was constrained by the energy of the first singlet excited state of Chl*a*, a constraint widely known as the ‘red‐limit’ (Bjorn *et al*., 2009). However, subsequent discoveries have unearthed oxygenic phototrophs that use different, red‐shifted Chl molecules (Miyashita *et al*., 1996; Chen *et al*., 2010; Gan *et al*., 2014; Antonaru *et al*., 2020), with less energy in their excited state, to catalyse the light reactions of oxygenic photosynthesis. The marine cyanobacterium *Acaryochloris marina* is one among them. More than 90% of the Chls within *A. marina* are Chl*d* (Miyashita *et al*., 1996, 1997), a pigment whose *Q*_Y_ absorption maximum is red‐shifted by *c*. 30 nm, relative to Chl*a*. The near ubiquity of Chl*d* in *A. marina* means that the cyanobacterium is well‐suited to living in shaded environments, in which visible light is filtered and far‐red light (FRL, 700–800 nm) is abundant (Kuhl *et al*., 2005). Strains of *A. marina* have been found in a variety of shaded marine environments, including the underside of ascidians (Kuhl *et al*., 2005; Lopez‐Legentil *et al*., 2011; Ohkubo & Miyashita, 2012) and within stromatolites (Johnson *et al*., 2022).

The red‐shifted nature of Chl*d* results in a *c*. 100‐meV loss of energy in its excited state compared with that of Chl*a*. Therefore, to efficiently make use of Chl*d*, the photosynthetic machinery of *A. marina* differs from that found in Chl*a*‐containing phototrophs (Elias *et al*., 2024). Differences are found in both its light‐harvesting antenna and its photosystems. Both PSI and PSII in *A. marina* use Chl*d* as their primary donors, which has implications for their ability to perform their catalytic chemistry. The primary donor of PSI, the Chl*d*/Chl*d*′ dimer, *P*_740_, is red‐shifted by 40 nm compared with that of the *P*_700_ dimer found in Chl*a*‐containing PSI of most cyanobacteria and plants (Hu *et al*., 1998). However, the midpoint potential of the *P*_740_^+^/*P*_740_ couple (425–440 mV) is essentially identical to that of the *P*_700_^+^/*P*_700_ couple (Bailleul *et al*., 2008; Schenderlein *et al*., 2008; Tomo *et al*., 2008), suggesting that it is the acceptor side of PSI that is altered to adjust for the loss of free energy in *P*_740_* (Petrova *et al*., 2023).

In the case of *A. marina* PSII, the nature of the primary donor is still debated (Mimuro *et al*., 2004; Itoh *et al*., 2007; Schlodder *et al*., 2007; Renger & Schlodder, 2008). The most widely accepted model of the initial charge separation steps proposes that the primary donor is Chl*d* (at the Chl_D1_ position), while Chl*a* occupies the P_D1_ position (Schlodder *et al*., 2007; Renger & Schlodder, 2008). Spectroscopic measurements have shown that this Chl absorbs at 725–727 nm, and it is consequently called *P*_727_ (Itoh *et al*., 2007; Schlodder *et al*., 2007). Like PSI, the primary donor of *A. marina* PSII has less energy in its excited state. By maintaining similar oxidative potentials on its donor side, PSII sacrifices energetic headroom on its acceptor side, a strategy that leads to enhanced recombination reactions (Viola *et al*., 2022).

Light harvesting in *A. marina* also occurs in an atypical manner compared with that in most cyanobacteria. In an approach similar to that used by light‐harvesting complex‐containing algae and plants, *A. marina* uses membrane‐bound, prochlorophyte Chl‐binding proteins (Pcb) to harvest light. Prochlorophyte Chl‐binding proteins are homologous to CP43, a core antenna protein of PSII, and the iron‐stress response protein, IsiA (Chen *et al*., 2005b; Murray *et al*., 2006), containing six transmembrane helices, which bind 14–19 Chls *d* and 3–4 zeaxanthins, depending on the specific Pcb isoform (Shen *et al*., 2024). Prochlorophyte Chl‐binding proteins in *A. marina* are bound to PSII, in which eight Pcb proteins bind to two PSII dimers, forming a PSII–Pcb supercomplex (Chen *et al*., 2005a; Shen *et al*., 2024).

Most *A. marina* strains do not possess phycobilisomes (PBS), with the notable exception of the well‐studied *A. marina* MBIC11017 (hereafter referred to as MBIC11017), which uses phycocyanin rods in addition to its Pcb antenna system. This increases the absorption capacity of MBIC11017 in the yellow‐orange spectral region, due to the PBS absorption maximum at *c*. 615 nm. Each phycocyanin rod consists of four stacked phycocyanin hexamers, with each monomer composed of an α‐and‐β subunit. This minimal PBS is markedly different from the large, hemidiscoidal PBS complexes found in many cyanobacteria (Bryant & Gisriel, 2024). It is thought that MBIC11017 acquired the genes required for the synthesis and degradation of its PBS system laterally, in order to expand its light‐harvesting capacity (Ulrich *et al*., 2021).

Regulation of PBS expression in MBIC11017 lies at the heart of its chromatic acclimation (CA) response to changes in light colour. When grown in orange light, MBIC11017 increases its PBS content, while growth in FRL leads to a decrease in its PBS content (Kashimoto *et al*., 2020). Growth in FRL also increases the transcript levels of a number of Pcb genes (Hernandez‐Prieto *et al*., 2018), suggesting that MBIC11017 changes its light‐harvesting antenna based on the availability and spectrum of incident light.

This acclimation response is analogous to the better studied far‐red light photoacclimation (FaRLiP) response (Gan & Bryant, 2015). Unlike *A. marina*, which is always capable of absorbing FRL due to its constitutive production of Chl*d*, FaRLiP cyanobacteria turn on their ability to harvest FRL, via the production of Chl*f* and Chl*d* and a red‐shifted PBS complex, as well as a number of FRL‐adapted photosynthetic protein subunits. Chl*f* is even more red‐shifted than Chl*d*, allowing FaRLiP cyanobacteria to absorb FRL up to 800 nm (Nurnberg *et al*., 2018; Mascoli *et al*., 2020). Furthermore, the FaRLiP response differentiates itself from *A. marina* by the number and placement of its red‐shifted pigments. FaRLiP cyanobacteria insert Chl*f* and Chl*d* into specific positions within each photosystem in which they account for < 10% of the total Chl content, while Chl*d* is present in nearly all positions of *A. marina* PSI and PSII.

It is still not fully understood why *A. marina* uses such a different strategy for far‐red photosynthesis compared with that for FaRLiP strains, although recent work suggests that there is a trade‐off between FRL absorption and resilience to photodamage, at least in regard to PSII (Viola *et al*., 2022). Additionally, it appears that even within the *A. marina* species, there have been functional diversifications of the light‐harvesting machinery, which most likely reflect adaptations to different light qualities and quantities (Ulrich *et al*., 2024).

In this work, we have taken a spectroscopic and biochemical approach to understand the bioenergetic implications of using Chl*d* as a major pigment for both light harvesting and photochemistry. Furthermore, we have investigated the role of the Pcb antenna of MBIC11017 in absorbing FRL, as well as the changes that occur to the photosynthetic machinery during MBIC11017's CA response. Combined, these results allow us to understand the spectral niche that MBIC11017 occupies and how this differentiates it from other *A. marina* strains.

## Materials and Methods

### Cell growth

*Acaryochloris marina* MBIC11017 (Miyashita *et al*., 2003) was obtained from the Biological Resource Center, NITE (NBRC; Japan). Cells were grown photoautotrophically in an IMK medium with 3.6% (w/v) artificial seawater (Aquaforest) at 25°C and at a constant irradiance of 20 μmol photons m^−2^ s^−1^ using either fluorescent tubes (WL) or 740‐nm LEDs (FRL). To ensure cells grown in WL or FRL were at the same stage of growth, they were either used directly in measurements or harvested for protein purification once they reached OD 0.4–0.6 at their *Q*_Y_ maxima.

### PSI isolation

Thylakoid membranes from MBIC11017 were isolated in a similar manner to that previously described (Chen *et al*., 2005a), with some minor changes which are described later. Cells were centrifuged (6500× ***g***, 15 min) and washed once in Buffer A (50 mM 2‐(N‐morpholino)ethanesulfonic acid (MES), 1 M betaine monohydrate, 5 mM CaCl_2_, 5 mM MgCl_2_ and 10% (v/v) glycerol; pH 6.5 adjusted with NaOH). Cells were centrifuged again and resuspended in Buffer A‐containing EDTA‐free protease inhibitor cocktail tablets (cOmplete; Roche), 0.2% (w/v) BSA and 50 μg ml^−1^ DNase I. Cells were lysed by three passages through a prechilled French pressure cell at 150 MPa and then centrifuged (2000× ***g***, 10 min, 4°C) to remove cell debris. Thylakoid membranes were pelleted by ultracentrifugation (190 000× ***g***, 30 min, 4°C) and resuspended in Buffer B (50 mM 2‐(N‐morpholino)ethanesulfonic acid (MES), 1 M betaine monohydrate, 20 mM CaCl_2_, 5 mM MgCl_2_ and 10% (v/v) glycerol; pH 6.5 adjusted with NaOH). Membranes were washed once in Buffer B and resuspended at a Chl*d* concentration of *c*. 1 mg ml^−1^.

Thylakoids were solubilised for 1 h in Buffer B containing 1% (w/v) n‐dodecyl‐β‐maltoside. Unsolubilised material was removed by centrifugation (17 000× ***g*** 10 min at 4°C). Solubilised thylakoids were loaded on a sucrose density gradient made by freezing and thawing 0.6 M sucrose, 50 mM MES‐NaOH at pH 6.5, 10 mM CaCl_2_, 5 mM MgCl_2_ and 0.04% (w/v) n‐dodecyl‐β‐maltoside buffer and separated by ultracentrifugation (270 000× ***g***, 15 h, 4°C). The PSI trimer band was collected and underwent a second round of sucrose density gradient and ultracentrifugation as specified previously (Supporting Information Fig. S1).

### Immunoblotting

Thylakoids at concentrations of 0.25, 0.5 and 1 μg of Chl were loaded onto a 12% tricine SDS‐Page Gel (Schagger, 2006). After electrophoresis, the proteins were transferred onto a nitrocellulose membrane using the *Trans*‐Blot Turbo Transfer System (Bio‐Rad), which was then blocked with 5% (w/v) milk in TBS‐T (20 mM tris (pH 7.6), 150 mM NaCl and 0.1% Tween 20) for 1 h and incubated with different primary antibodies overnight in the dark at 4°C. Primary antibodies were diluted in the following ratios: PsaB (1 : 5000); CP47 (1 : 10 000); and IsiA (1 : 1000). All antibodies were purchased from Agrisera. After incubation, the membrane was washed 3 × 5 min in TBS‐T and incubated with the secondary antibody (goat anti‐rabbit immunoglobulin G) for 1 h. The membrane was washed with 3 × 5 min in TBS‐T and developed for chemiluminescence (with agent SuperSignal West Pico; Thermo Fisher Scientific, Waltham, MA, USA) using a LAS 4000 Image Analyzer (ImageQuant). Densitometry analysis was performed using the imagej software. Blots can be seen in Fig. S2.

### PSII antenna size measurements

Photosystem II antenna size measurements were made using the fluorescence induction method with a JTS‐10 spectrophotometer (BioLogic) as described previously (Tian *et al*., 2019). Cells were dark‐adapted for 5 min before the addition of 50 μM 3‐(3,4‐dichlorophenyl)‐1,1‐dimethylurea (DCMU). Actinic light was provided at subsaturating intensities using 630 or 730‐nm LEDs, and fluorescence was detected using a weak WL LED filtered through a 440‐nm filter (10 nm FWHM, Schott).

In the presence of DCMU and subsaturating actinic light, PSII fluorescence rises to a maximum (*F*_M_), which corresponds to a single‐charge separation event in all centres. The rate of this fluorescence rise (i.e. the PSII electron transport rate (ETR)) is proportional to both the actinic light intensity and the PSII functional antenna size. In order to obtain the PSII ETR, the reciprocal of the area above the fluorescence induction curve after the first 120 ms of measuring was calculated. This was then plotted as a function of the light intensity. By performing a linear regression (scikit‐learn) forced through the origin (0,0), the functional PSII sizes could be obtained as the slopes of the fits.

### Steady‐state spectroscopy

Absorption spectra were recorded using a Varian Cary 4000 UV‐VIS spectrophotometer. For measurements on cells, an integrating sphere module was used. Absorption spectra at 77 K were performed using a custom‐built liquid nitrogen cooling device. For these measurements, the sample was supplemented with 70% (v/v) glycerol to prevent the formation of ice crystals. The emission and excitation spectra were recorded on a HORIBA JobinYvon‐Spex Fluorolog 3.22 spectrofluorimeter using a sample concentration of OD < 0.05 cm^−1^ at the *Q*_y_ maximum. For the 77‐K emission spectra, the samples were measured in a 1‐mm path‐length Pasteur pipette frozen in liquid nitrogen.

### Streak camera

The streak camera set‐up has been previously described in detail (Hu *et al*., 2023). Excitation pulses were focussed to a spot size of *c*. 100 μm. For measurements of cells with PSII in the open state, a flow‐cuvette with a flow speed of 2.5 ml s^−1^ and excitation powers of 5 μW (400 nm) or 10 μW (580 nm) was used. For measurements of cells with PSII in the closed state, a magnetically stirred 1 × 1 cm cuvette with 50 μW excitation power was used. Additionally, 50 μM DCMU was added to the cells, which were pre‐illuminated in room light before the measurement. For the measurements of isolated PSI, excitation powers of either 50 μW (RT) or 150 μW (77 K) at 400 nm were used. Additional details can be found in Methods S1.

### TCSPC

The time‐correlated single photon counting (TCSPC) measurements were taken with a PicoQuant FluoTime 200. A laser diode running at 10 MHz and a centre wavelength of 440 nm provided the excitation light. The instrument response function was measured by recording the fluorescence decay of pinacyanol iodide in methanol, which has a fluorescence lifetime of 6 ps (Van Oort *et al*., 2008), and it had a determined full width half maximum of 88 ps. The sample concentrations were < 0.3 cm^−1^. The samples were constantly stirred with a stir bar revolving at 1000 rpm. For the measurements on living cells in open PSII conditions, power studies were performed to make sure that PSII was in the open state (Fig. S3). For the measurements on the cells in closed PSII conditions, 50 μM of DCMU was added, the samples pre‐illuminated and the power increased to 66 μW.

### Global and target Analysis

The global analyses for the streak camera data were performed using the pyglotaran python package (van Stokkum *et al*., 2004, 2023). The fits obtained by the global analyses of the streak camera data can be seen in Figs S4–S12. The global analyses for the TCSPC data were performed with the TRFA Data Processor Advanced software (Digris *et al*., 2014). The fits obtained by the global analyses of the TCSPC data can be seen in Figs S13–S18.

A target analysis was performed on the streak camera data of the cells in open PSII conditions excited at 400 and 580 nm, and of the isolated PSI complex, simultaneously, using the pyglotaran python package (van Stokkum *et al*., 2023). The kinetic scheme used is shown in Fig. 5(a). The fits obtained by the simultaneous target analysis of the streak camera data can be seen in Figs S19 and S20. Additional details can be found in Methods S2.

### Excitation energy trapping simulations

Excitation energy trapping dynamics within the PSII was simulated as described previously (Gradinaru *et al*., 1998; Amerongen & Grondelle, 2000; Croce & van Amerongen, 2020) in which the excitation energy transfer (EET) rates between the Chls are calculated based on the Förster resonance energy transfer theory. The coordinates of the chlorins were extracted from the Protein Data Bank with entry 7YMM (Shen *et al*., 2023). Additional details can be found in Methods S3.

## Results

### Chromatic acclimation

**Fig. 1:**
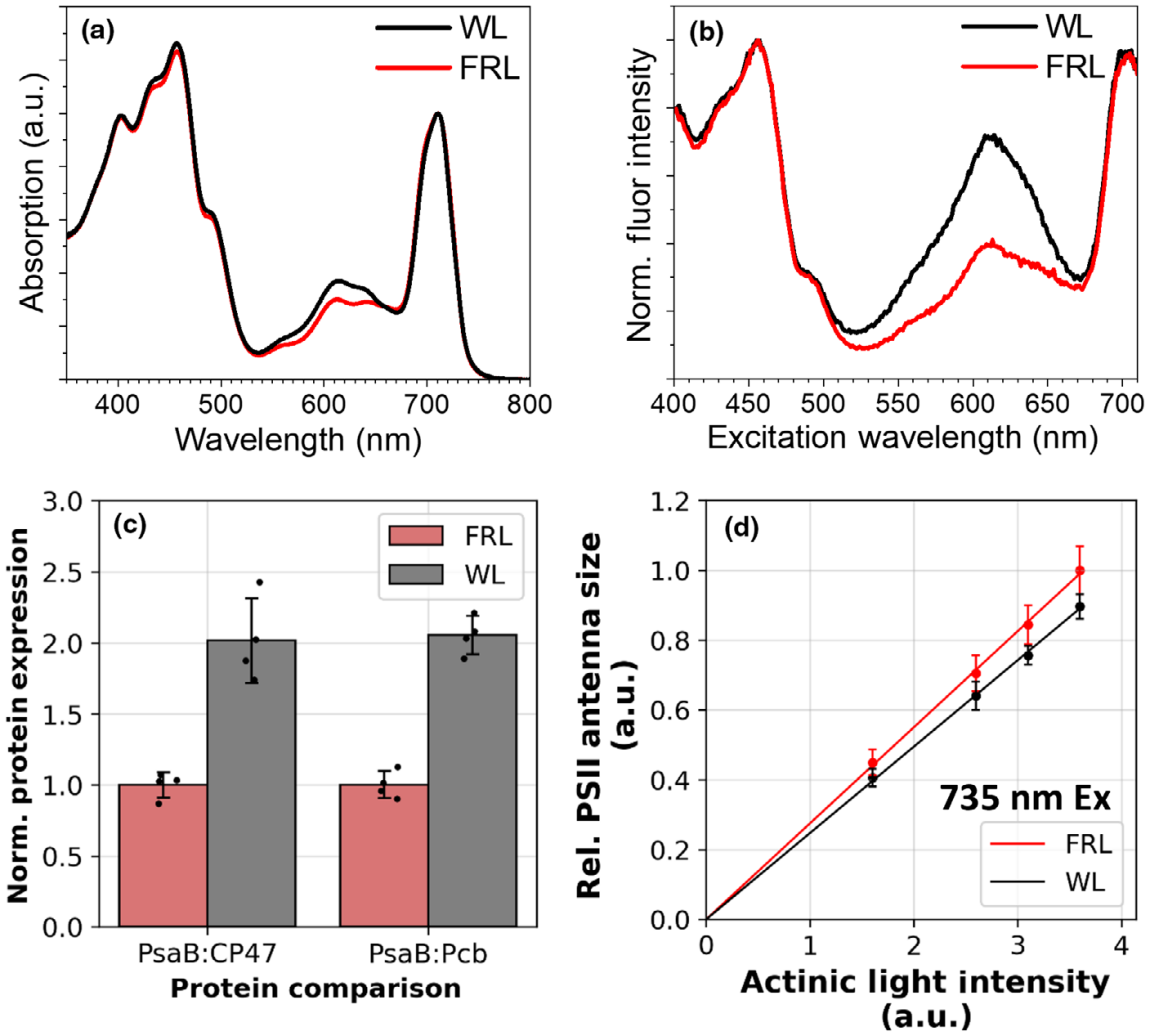
Chromatic acclimation of *Acaryochloris marina* MBIC11017 to growth in white light (WL) or far‐red light (FRL). (a) Absorption spectra of the cells grown in WL and FRL. Spectra were normalised to their maxima at *c*. 710 nm. (b) Excitation spectra of the cells monitored at 725 nm. Spectra were normalised to their maxima at *c*. 710 nm. 3‐(3,4‐Dichlorophenyl)‐1,1‐dimethylurea (DCMU) was added to the cells to close Photosystem II (PSII). Considering that the lifetime of PSII in its closed state is orders of magnitude longer than Photosystem I (PSI) (*vide infra*), these excitation spectra can be regarded as PSII action spectra. (c) Relative protein abundance comparison as determined by immunoblotting. Signal intensities were normalised to the amount of PsaB present in FRL samples. The bar graphs show the mean ± SD, with individual data points overlaid. (d) Relative PSII antenna size of WL and FRL cells after excitation at 735 nm. Antenna sizes have been normalised relative to the maximum PSII antenna size measured in FRL cells. Error bars show SD.

To understand the changes that occur during the MBIC11017 CA response, we grew cells in either WL (fluorescent tubes) or FRL (*c*. 740‐nm LEDs). The absorption spectra of WL‐ or FRL‐grown cells (Fig. 1a) are identical in the *Q*_y_ region and very similar in the Soret region, indicating that no different spectral forms within the photosystems or Pcb antenna are synthesised between the two conditions, and the carotenoid composition does not drastically change. The main difference is in the 560‐ to 640‐nm region, where the amplitude is notably smaller in the FRL‐grown cells. Absorption in this region is due to phycocyanin, showing that the PBS content, relative to Chl*d*, in FRL‐grown cells is less than in WL cells, as a result of MBIC11017 CA. This difference in the relative PBS content is even larger when comparing WL and FRL MBIC11017 PSII via their excitation spectra (Fig. 1b). Considering that for both growth conditions, the amplitude of the PBS peak vs the *Q*_y_ peak in the PSII excitation spectrum is higher than in the corresponding absorption spectrum (Fig. 1a), we can conclude that there are more PBS connected to PSII than to PSI (clarified in more detail by the time‐resolved fluorescence (TRF) measurements later).

We probed the effect of MBIC11017 CA on photosystem and Pcb abundances by performing immunoblot analysis of thylakoids from WL‐ and FRL‐grown MBIC11017. Photosystem I accumulation was inferred using a PsaB antibody, while a CP47 antibody was used for PSII. In WL, the relative abundance of PsaB to CP47 was twice that in FRL (Fig. 1c). Similarly, WL thylakoids exhibited a relative abundance of PsaB to Pcb antenna that was twice that in FRL thylakoids (Pcb proteins were probed using an IsiA antibody known to recognise Pcb proteins; Li *et al*., 2018).

To assess whether these CA changes extended to a functional level, the functional PSII antenna size of WL‐ and FRL‐grown cells was measured using the fluorescence induction method (Tian *et al*., 2019). Fig. 1(d) shows the PSII ETRs of WL‐ and FRL‐grown cells when exposed to 735‐nm actinic light. The 735‐nm actinic light was used to selectively excite Chl*d* within both the PSII core and the Pcb antenna (rather than excitation of PBS). The resulting actinic light vs ETR slope is slightly larger for FRL cells than for WL‐grown cells, indicating a *c*. 10% larger Chl*d*‐based PSII antenna size, although this value lies within the margin of error. A similar measurement, using a 630‐nm actinic light, indicates that WL‐grown cells have a larger PBS‐associated PSII antenna size (Fig. S21).

**Fig. 2:**
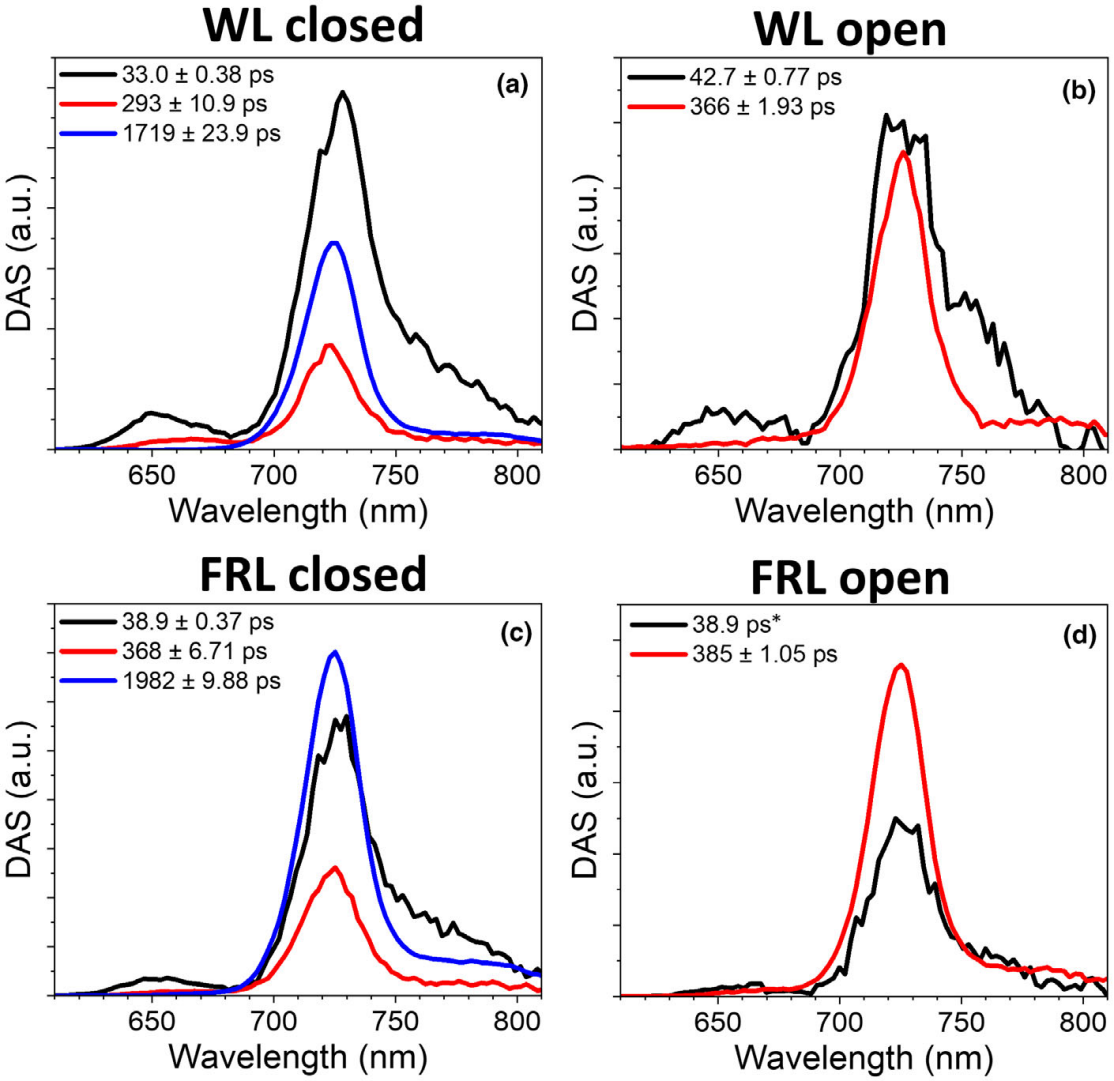
Global analysis of time‐resolved fluorescence data from *Acaryochloris marina* MBIC11017 cells grown in white light (WL) or far‐red light (FRL). Decay‐associated spectra (DAS) of cells with Photosystem II in either the closed state (a, c) or in the open state (b, d). Excitation was provided at 400 nm. The asterisk indicates a fixed lifetime component. Both lifetimes and SE are displayed.

Time‐resolved fluorescence measurements were taken on WL‐ and FRL‐grown cells to understand how EET dynamics change as a result of MBIC11017 CA. Using a streak camera set‐up, cells were excited at 400 nm to preferentially excite Chl*d* within the photosystems and Pcb antenna. High excitation power and DCMU were used to keep PSII in its closed state. This increases its emission lifetime, allowing for better disentanglement of the PSI contribution. Global analysis of WL‐ and FRL‐grown cells in the closed state is shown in Fig. 2(a,c). The excitation energy trapping dynamics within WL cells could be satisfactorily described by three components. The first component, which shows a larger amplitude *c*. 750 nm than the other two and has a 33‐ps lifetime, is attributed to PSI. The remaining two components, which are spectrally almost identical to one another, have longer lifetimes of *c*. 300 ps and 1.7 ns and are attributed to PSII. Far‐red light‐grown cells also exhibit three energy trapping components, which share similar lifetimes (38.9 ps, 368 ps and 1.98 ns) and spectra to the WL‐grown cells. However, the combined amplitude of the PSII‐associated components relative to the PSI component is about two times larger in FRL than in WL, indicating a large difference in PSI/PSII ratio, consistent with the immunoblotting results (Fig. 1c).

The measurements were repeated in WL and FRL cells but using a flow cell cuvette and lower excitation powers in order to keep PSII in an open state. Global analysis of these measurements is shown in Fig. 2(b,d). The lifetime of PSII in its open state is 366 ps for WL cells and only slightly longer in FRL cells (386 ps).

### Excitation energy trapping in PSI

**Fig. 3:**
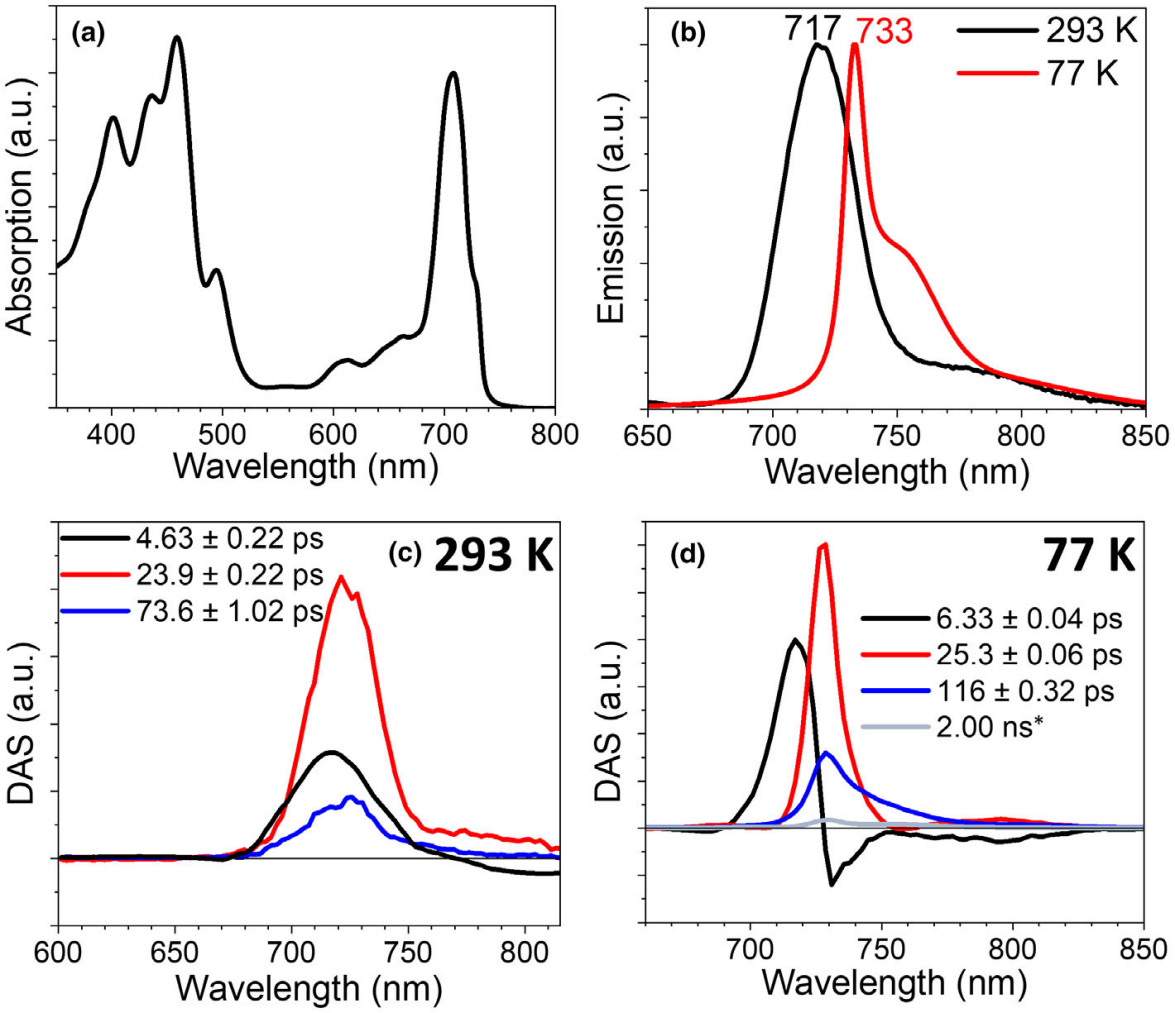
Spectroscopic characterization of *Acaryochloris marina* MBIC11017 Photosystem I (PSI). (a) 77‐K absorption spectrum of MBIC11017 PSI. (b) Room temperature and 77‐K emission spectra of Photosystem I (excited at 500 nm). The spectra were normalised to their maxima. (c, d) Decay‐associated spectra (DAS) for the streak camera measurement on PSI excited at 400 nm at room temperature (c) and 77 K (d). The asterisk indicates a fixed lifetime during the global analysis fitting. Both lifetimes and SE are reported. The 293‐K DAS and the 77‐K emission spectrum have been smoothed.

To observe the MBIC11017 EET dynamics in finer detail, PSI was isolated from WL‐grown cells. The 77‐K absorption spectrum (Fig. 3a) is virtually identical to previously reported spectra (Tomo *et al*., 2008). The main peak in the *Q*_y_ region is located at 708 nm. There is a distinct shoulder *c*. 728 nm, and the spectrum skews at 740 nm. The distinct bands indicate the presence of multiple spectral pools of Chls *d* in the PSI complex. A Gaussian deconvolution was performed on the *Q*_y_ region of the spectrum (Fig. S22A). Considering 100 Chls per PSI (Tomo *et al*., 2008; Elias *et al*., 2024), the deconvolution reveals that the shoulder at 728 nm corresponds to 22.3 Chls *d* and that the skewing *c*. 740 nm can be described by two Gaussians together amounting to *c*. 1.7 Chls *d*. The emission spectra of MBIC11017 PSI are shown in Fig. 3(b). At room temperature, there is one main broad *Q*_y_ emission band peaking at 717 nm. The broadness of the band suggests that multiple spectrally different pools of Chl*d* are emitting. At 77 K, the main emission peak narrows and shifts to 733 nm, and a distinct shoulder *c*. 755 nm emerges.

To assess which absorption bands contribute to which emission bands, we have calculated the 77‐K emission spectrum from the Gaussian deconvolution of the 77‐K absorption spectrum, taking into account a Stokes shift of 100 cm^−1^ and by calculating the excited state population of all the Gaussian bands using the Boltzmann equation, taking their centre position as their energy and their relative area as their relative abundance. This analysis reveals that the 755‐nm shoulder in the emission spectrum can be attributed to the Chls *d* absorbing *c*. 740 nm (Fig. S22B). However, based on this analysis, one would expect to see hardly any emission at 733 nm. When the same analysis is applied excluding the two lowest energy Gaussians, the calculated emission profile replicates the 733‐nm band (Fig. S22C), revealing that the Chls *d* absorbing *c*. 728 nm are responsible for this emission band. These results suggest that these pigment pools are not functionally connected. If they were part of the same complex, we would expect energy equilibration, yet this is not observed. The persistence of fast trapping even at low temperature (will be discussed later) suggests that excitation energy is being utilised within separate pools rather than equilibrating between them, which could be explained by the presence of distinct conformational states.

To investigate the PSI trapping dynamics, we performed streak camera measurements at room temperature using 400‐nm excitation. The TRF data were globally analysed, and the resulting DAS are shown in Fig. 3(c). The first component has a lifetime of 4.6 ps and shows positive signals peaking *c*. 710 nm and negative signals above 750 nm, which indicate ultrafast EET processes. Since the integrated spectrum is positive, we cannot exclude that some trapping already occurs within this time window. The main decay component is described by the second component, which has a lifetime of 23.9 ps. Part of the excitation energy is trapped on a slower timescale of 73.6 ps, as seen in the last component. The second and third DAS are similar, although the 73.6‐ps DAS are slightly larger in amplitude *c*. 750 nm (Fig. S23).

To verify that this slower component was *bona fide* PSI trapping, and not caused by PSII contamination, we repeated the measurement in the presence of DCMU, which should increase the lifetime of PSII but not PSI. No changes were observed, indicating that this component could be attributed to PSI (Fig. S24). In addition, in a previous transient absorption study on MBIC11017 PSI, a slow component of 71 ps was observed after excitation at 630 nm (Petrova *et al*., 2023).

To assess the possible contribution of red‐shifted Chl pools on the trapping dynamics, we measured MBIC11017 PSI at 77 K with 400‐nm excitation. Four components were extracted from the global analysis, and DAS are shown in Fig. 3(d). The first DAS (6.33 ps lifetime) has positive/negative features showing EET from high‐energy Chls *d* (720 nm) to low‐energy ones (peak at 733 nm) and extending up to 800 nm. The second component has a lifetime of 25.3 ps, and its spectrum is positive, with a sharp peak centred at 733 nm. The third component has a lifetime of 116 ps and also represents a decay of Chl*d* excited states. Its amplitude is about one‐sixth of the 25.3‐ps component, and its spectrum has a larger amplitude in the red region. Remarkably, trapping is relatively fast even at 77 K: In comparison, in *Synechocystis* sp., PCC 6803 PSI trapping from the lowest energy forms takes 1.14 ns at 77 K (Akhtar *et al*., 2021). The dynamics of the MBIC11017 PSI at 77 K are resemblant of those of *Synechococcus* WH 7803 PSI, which is devoid of red forms (van Stokkum *et al*., 2013), and in which there is also one small trapping component with a slightly red‐shifted spectrum and a lifetime of 140 ps (Acuna *et al*., 2018). These data indicate that there are only a few low‐energy forms in MBIC11017 PSI.

### Excitation energy trapping in PBS

**Fig. 4:**
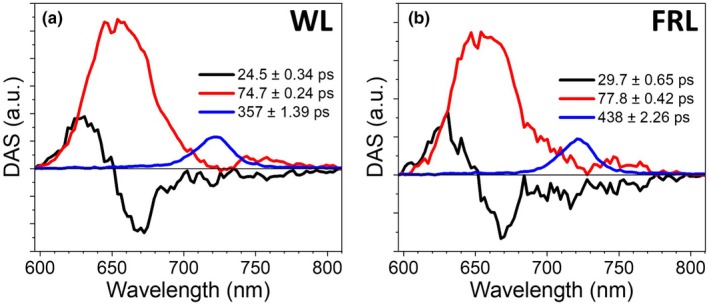
Global analysis results of the streak camera measurements of *Acaryochloris marina* MBIC11017 cells upon phycobilisome excitation. Decay‐associated spectra (DAS) of (a) white light (WL)‐ or (b) far‐red light (FRL)‐grown cells with Photosystem II in the open state. The excitation was provided at 580 nm. Both lifetimes and SE are displayed.

To get insight into the EET dynamics within PBS complexes and between them and the photosystems, the cells grown in the two light conditions were measured using a 580‐nm laser excitation. To keep PSII in its open state, low excitation densities were used, and the cells were continuously flowed through a flow‐cuvette. The global analyses of these data sets are presented in Fig. 4. For both the WL and FRL data sets, three components were sufficient to satisfactorily fit the data. All three components are very similar across the two data sets in both spectrum and lifetime. The first component has a 24.5/29.7 ps (WL/FRL) lifetime and a conservative spectrum. It describes EET within the PBS from phycocyanobilins, which have emission *c*. 630 nm, to phycocyanobilins emitting *c*. 680 nm. The second component has a lifetime of 74.7/77.8 ps (WL/FRL) and is positive. The spectrum peaks *c*. 650 nm and mainly describes the depopulation of phycocyanobilin excited states. In both DAS, a clear dip *c*. 725 nm can be observed, which indicates EET to Chls *d*. The final component for both data sets has the spectrum of Chls *d* and a lifetime that corresponds to PSII in its open state. The lifetime of this component is longer for the cells grown in FRL (438 ps) than those in WL (357 ps), in agreement with the longer PSII lifetime of the cells grown in FRL (Fig. 2a,c), or possibly we did not succeed in keeping PSII fully in its open state in the measurement on the FRL cells. It is readily observed that the area that corresponds to the depopulation of the PBS excited states in the second DAS is much larger than the dip that corresponds to the excitation energy acceptor Chls *d*. On the one hand, this can be explained by the fact that the oscillator strength of the phycocyanobilins is several times larger than that of Chls *d* (Notes S1). On the other hand, this phenomenon could also be explained by part of the PBS transferring energy to PSI, resulting in inverted kinetics as the PSI complex is depopulated on a similar timescale as populated.

**Fig. 5:**
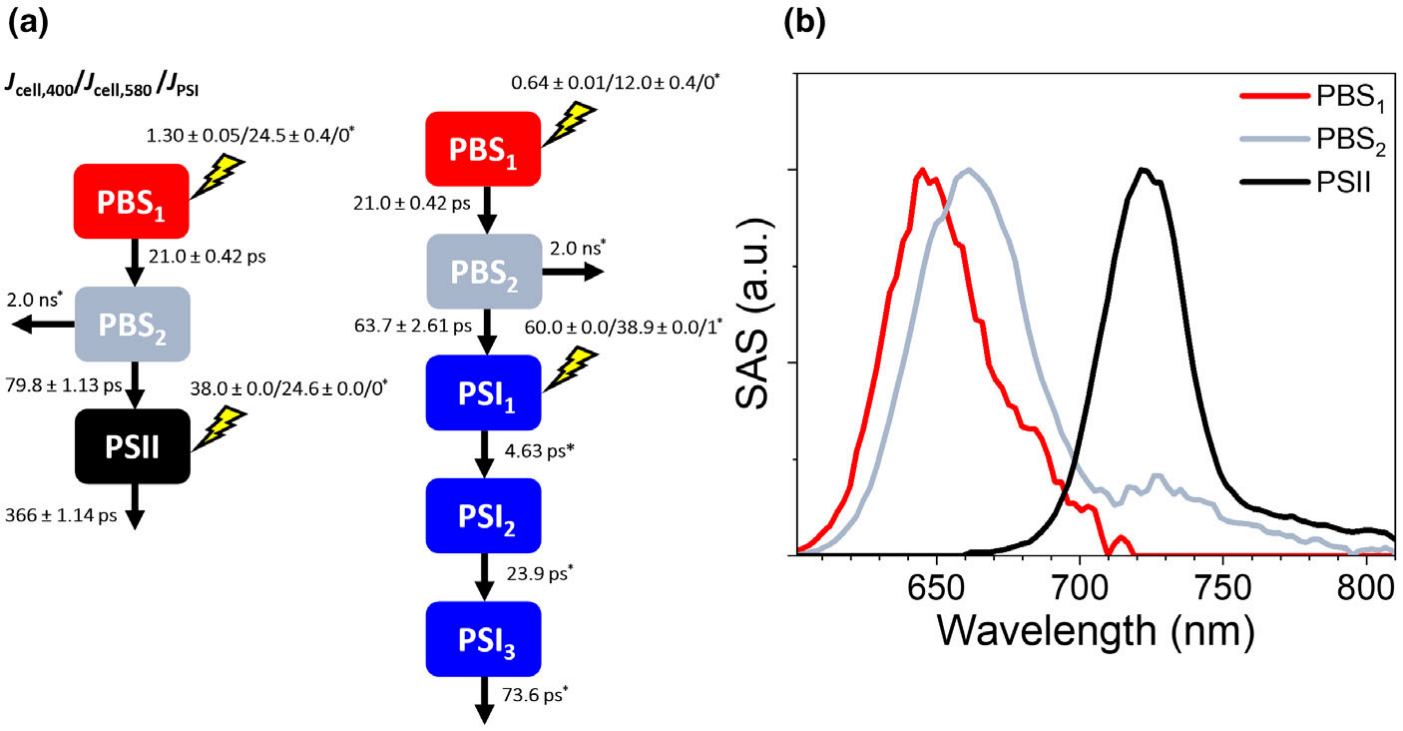
Target analysis fitting results of streak camera measurements of *Acaryochloris marina* MBIC11017 cells grown in white light after excitation at 400 nm or 580 nm. (a) The kinetic scheme used for the target model showing the fit parameters (lifetimes and initial excitation vectors) and their associated SE. The phycobilisome (PBS) dynamics were described by two compartments (PBS_1_ and PBS_2_). Photosystem II (PSII) and Photosystem I (PSI) dynamics were described by one (PSII) and three (PSI_1_, PSI_2_ and PSI_3_) compartments, respectively. The lightning bolts above each of the sequential branches in the kinetic scheme represent the initial excitation vector. Asterisks indicate a fixed parameter. (b) Normalised species‐associated spectra of PBS_1_, PBS_2_ and PSII from the target analysis.

To better understand the EET dynamics from the PBS to the photosystems, we performed a target analysis for WL‐grown cells excited at 580 and 400 nm, and the isolated PSI complex simultaneously. The full kinetic scheme is shown in Fig. 5(a) and described in detail in the Materials and Methods section. In short, the PSI dynamics were fit with the components arising from the analysis on the isolated complex to allow for a better disentanglement of the PBS and PSII dynamics. The trapping of excitation energy by PSII is described with a single compartment, whereas PBS is described with two compartments, as suggested by global analysis (Fig. 4). We modelled two separate PBS pools, one that transfers excitation energy to PSII and another to PSI. The kinetic scheme and fitted parameters of the target analysis are shown in Fig. 5(a) and the normalised species‐associated spectra in Fig. 5(b).

The analysis confirms that there are PBS connected to both PSI and PSII, with about two‐thirds connected to the latter. This is in agreement with the observation that in the PSII excitation spectrum (Fig. 1b), the PBS peak is significantly larger than in the corresponding absorption spectrum (Fig. 1a). These data are also in agreement with the PSI and PSII action spectra reported by Boichenko *et al*. (2000). However, it should be noted that the precise distribution of PBS connected to PSI and PSII depends strongly on the chosen relative oscillator strength of the phycocyanobilins compared with that of the Chls *d*; that is, the ratio of PBS connected to PSII vs PSI increases with an increasing oscillator strength of the phycocyanobilins. However, we can exclude that PBS only transfer to PSII, since only once a nine times larger oscillator strength of the phycocyanobilins over Chl*d* is assumed, then the model predicts that practically all of the PBS excitation energy is transferred to PSII. The target model moreover reveals that the PBS transfer their excitation energy somewhat faster to PSI (τ = 63.7 ps) than to PSII (τ = 79.8 ps).

### Excitation energy trapping in PSII

**Fig. 6:**
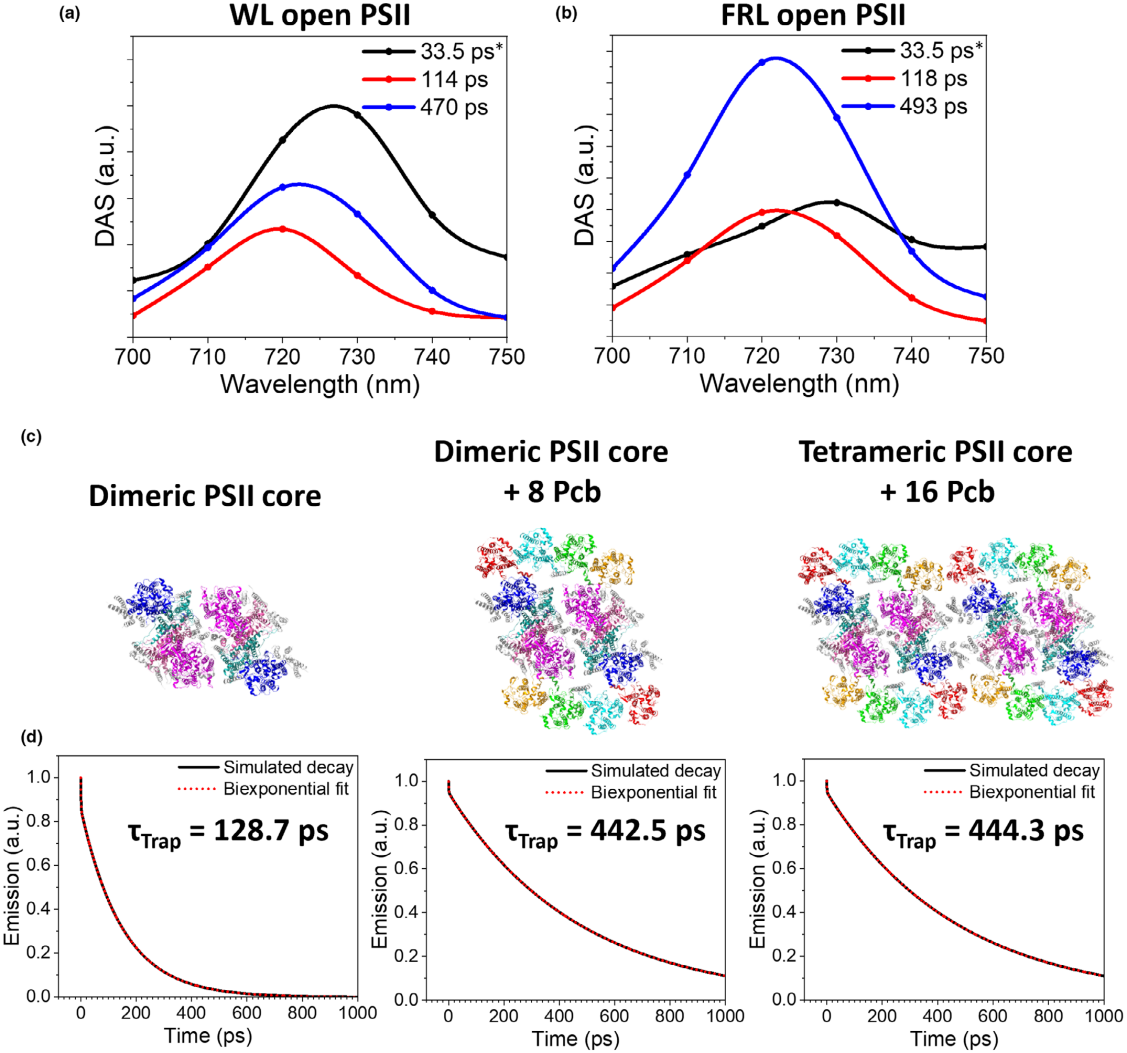
Global analysis of time‐resolved fluorescence (TRF) data from *Acaryochloris marina* MBIC11017 cells and simulation of Photosystem II (PSII) trapping dynamics. Global analysis of TRF data from (a) white light (WL)‐ and (b) far‐red light (FRL)‐grown MBIC11017 cells with PSII in its open state, obtained using the time‐correlated single‐photon counting set‐up. Excitation was provided at 440 nm. Asterisks indicate a fixed lifetime component. (c) Structures used for energy trapping simulations of PSII (PDB: 7YMM; Shen *et al*., 2023). (d) The simulated trapping dynamics (solid lines) are shown in combination with a biexponential fitted curve (red dotted lines). The average trapping time (τ_Trap_) for each simulation is also reported (see Supporting Information Table S1 for the amplitude and lifetimes for the biexponential fit).

To further investigate the trapping dynamics within PSII, TCSPC measurements have been taken on the cells grown in the two light conditions. A 440‐nm excitation wavelength was used, which practically exclusively excites the photosystems, as can be observed from the 440‐nm cell emission spectrum (Fig. S25). Compared with the streak camera set‐up, the TCSPC set‐up has a superior signal‐to‐noise ratio (SNR) and allows for a longer measurement time window, whereas the streak camera has a better temporal‐spectral resolution. In addition, the superior SNR of the TCSPC set‐up allowed for a careful power study to ensure that PSII is in its fully open state (Fig. S3A,B). The global analysis results of the TCSPC measurements are shown in Fig. 6(a,b). Since the PSI dynamics are better resolved with the streak camera, we have fixed the lifetime of the first component during the TCSPC global analysis to the average lifetime of the isolated PSI complex (33.5 ps). The PSII dynamics were consequently fit with the remaining components and were resolved to be biphasic. The PSII trapping had similar time constants in both light conditions (114 and 470 ps for WL cells vs 118 and 493 ps for FRL cells), but differed in relative amplitude, giving rise to an average trapping time of 330 ps for WL cells and 376 ps for FRL cells. These average PSII lifetimes are close to those observed with the streak camera (Fig. 2a,c), showing that the PSII was in its open state also in those measurements.

The second PSII component is relatively long, considering that Chl*a*‐containing PSII traps excitation energy in the order of 60–100 ps (Croce & van Amerongen, 2020). As such, we sought to understand the origin of this long trapping component by performing additional TRF measurements on *Prochlorococcus marinus* MIT9301 (hereafter referred to as *Prochlorococcus*). A very similar low‐light‐adapted ecotype of *Prochlorococcus* has been shown to assemble PSII‐Pcb supercomplexes (Bibby *et al*., 2003), which share an identical supramolecular structure to the PSII‐Pcb supercomplexes present in MBIC11017 (see Fig. S26; Notes S2; Methods S4 for an in‐depth explanation). Since *Prochlorococcus* uses the high‐energy divinyl Chl*a* rather than Chl*d*, the effect of the larger antenna system in PSII‐Pcb supercomplexes on its trapping time can be differentiated from any possible influence by Chl*d*.

The results of the global analysis of the *Prochlorococcus* TRF measurements are shown in Fig. S27. The TCSPC and streak camera measurements show a 34–41 ps trapping time for PSI. In the streak camera measurements, a single PSII lifetime of 374 ps was resolved, which could be disentangled into two components of 97 and 464 ps in the TCSPC measurements. This biphasic behaviour, and its associated lifetimes, is remarkably similar to that seen in MBIC11017 cells.

Recently, Shen *et al*. (2024) resolved structures from MBIC11017 of a PSII dimer and a PSII tetramer, both of which were flanked by Pcb proteins. We could then simulate the EET dynamics based on the resolved PSII structures for three different structural scenarios: the MBIC11017 PSII dimeric core (with no Pcb antenna), the PSII‐Pcb dimer and the PSII‐Pcb tetramer (see the Materials and Methods section for details). The simulated trapping dynamics and their bi‐exponential fits for each scenario are presented in Fig. 6(c,d) (see Table S1 for the simulated amplitudes and lifetimes). The simulated trapping time for the PSII dimeric core (129 ps) is similar to the experimentally resolved fast trapping time (*c*.115 ps). In the case of the PSII‐Pcb dimer and the PSII‐Pcb tetramer, the simulated trapping times were identical to one another (443 and 442 ps, respectively) and very similar to the slow component resolved in the TCSPC experiments (*c*. 480 ps). Combined, these results indicate that the slow PSII trapping component in MBIC11017 (and *Prochlorococcus*) is due to the presence of Pcb antenna proteins, which slow down energy migration to the PSII core.

## Discussion

The use of FRL for photosynthesis by oxygenic phototrophs is a challenge due to the energetic constraints imposed by the catalytic chemistry of each photosystem (Elias *et al*., 2024). This is exemplified in PSII, in which the reduced primary donor energy and high energetic requirement of water oxidation lead to altered acceptor side energetics in MBIC11017 and the Chl*f*‐containing PSII from the FaRLiP cyanobacterium, *Chroococcidiopsis thermalis* (Viola *et al*., 2022). Furthermore, the use of red‐shifted pigments for light harvesting can also provide a challenge. These red‐shifted pigments can act as low‐energy traps, increasing the time of energy migration to the reaction centre and lowering the quantum efficiency of charge separation. We begin our discussion by examining the molecular mechanisms used by MBIC11017 in order to efficiently harvest FRL in each photosystem, before moving on to its CA response, which allows it to adapt to WL, despite its FRL specialism.

### Excitation energy trapping in PSI

The trapping of excitation energy within the isolated MBIC11017 PSI occurs within 33.5 ps, which equates to a charge separation efficiency (φ_CS_) of *c*. 98% (φ_CS_ = 1–τ_CS_(33.5 ps)/τ(2 ns) = 0.98, where τ_CS_ is the photosystem trapping lifetime and τ(2 ns) is the excited state lifetime of Chl in the absence of photochemistry (Belgio *et al*., 2012)). This trapping time is virtually the same as the measured *in vivo* trapping time of the cells grown in WL (33.0 ps, Fig. 3b), and slightly shorter than the *in vivo* PSI trapping time in FRL‐grown cells (38.9 ps, Fig. 3d). These data indicate that the isolated PSI complex maintains its native architecture upon isolation. The MBIC11017 PSI trapping time is moreover strikingly close to the *in vivo* PSI trapping time of *Prochlorococcus* cells (35.8 ps, Fig. S27B). Like the MBIC11017 PSI, the *Prochlorococcus* PSI spectrum contains slightly more amplitude in the red than its corresponding PSII spectrum (Fig. S27D). Given these similarities, we argue that the MBIC11017 PSI trapping time is not greatly affected by the presence of Chls *d* in the reaction centre (RC).

A striking feature of MBIC11017 PSI is the near‐total absence of canonical red forms, that is Chls absorbing at energy lower than the reaction centre (Karapetyan *et al*., 2006). This is evident from the very short decay time of the most red‐shifted component at 77 K, indicating efficient energy trapping in the reaction centre. This is at variance with the PSI of plants and other cyanobacteria, where at 77 K, the decay occurs on the nanosecond timescale due to the trapping effects of the red forms (Croce *et al*., 2000).

### Excitation energy trapping in PSII

Excitation energy trapping in dimeric PSII cores typically occurs in 60–100 ps (Croce & van Amerongen, 2020). However, our results show that trapping in MBIC11017 PSII is far slower (330–380 ps) and biphasic, with a shorter component of *c*. 115 ps and a longer component of *c*. 480 ps. The trapping time in photosystems is a combination of the time it takes for energy to migrate to the reaction centre (migration time) and the rate at which charge separation occurs (charge separation time) (Croce & van Amerongen, 2011). As such, there are two possibilities that can explain why PSII trapping is slowed in MBIC11017. First, the presence of Chl*d* in the PSII RC (or associated redox tuning effects of the RC cofactors) could result in a slowed rate of the primary charge separation processes. Second, the increased number of Chls associated with the MBIC11017 PSII‐Pcb supercomplex (relative to dimeric PSII cores) could increase its energy migration time. Of course, a combination of these two effects may also be a possibility.

In MBIC11017 PSII, Chl*d* is used as the primary electron donor (Schlodder *et al*., 2007; Renger & Schlodder, 2008), which results in a loss of *c*. 100 meV in its excited state compared with *P*_680_* in Chl*a* PSII. In the primary steps of PSII charge separation, Pheo_D1_ is reduced, forming Chl_D1_^+^Pheo_D1_^−^, and the P_D1_ Chl is oxidised, forming P_D1_^+^Pheo_D1_^−^. Therefore, if the redox potential of the Pheo_D1_/Pheo_D1_^−^ couple was the same in MBIC11017 PSII as in Chl*a* PSII, the driving force for the formation of the primary charge‐separated states would be significantly diminished. However, redox titrations of MBIC11017 PSII cores have shown that *E*_m_ (Pheo_D1_/Pheo_D1_^−^) is *c*. 125 mV more positive than that in Chl*a* PSII from cyanobacteria (−401 vs −525 mV, respectively) (Sugiura *et al*., 2010; Allakhverdiev *et al*., 2011). This upshift in the pheophytin redox potential means that the driving force for the formation of the secondary radical pair (P_D1_^+^Pheo_D1_^−^) is roughly the same in MBIC11017 PSII as in Chl*a* PSII (Viola *et al*., 2022), and the rate of charge separation should also be roughly the same.

*Prochlorococcus* does not possess Chl*d* (instead it uses the higher energy divinyl Chl*a* and Chl*b*), yet it still exhibits a long PSII trapping lifetime (374 ps, Fig. S27A), suggesting that the long trapping lifetime in MBIC11017 PSII is not due to the presence of Chl*d*. Therefore, the longer PSII trapping lifetime observed in MBIC11017 PSII should result from a longer energy migration time. Both conventional electron microscopy (Chen *et al*., 2005a) and cryoEM (Shen *et al*., 2024) have shown that the MBIC11017 PSII‐Pcb supercomplex possesses four Pcb proteins (binding 69 Chls *d*) per PSII core (which binds 37 Chls *d*). As a result, the PSII‐Pcb supercomplex antenna size is nearly three times larger than the PSII core, increasing its excitation energy migration time compared with cores alone. *Prochlorococcus marinus* MIT9313 possesses a PSII‐Pcb supercomplex with an identical architecture to the one in MBIC11017 (Bibby *et al*., 2003). As the PSII trapping times in *Prochlorococcus* are highly similar to MBIC11017 and also biphasic, it suggests that the PSII antenna configuration, rather than the Chl type, determines these trapping times.

This finding was confirmed by simulating the MBIC11017 PSII trapping times based on the cryoEM structure (Fig. 6). Not only are these simulated lifetimes highly similar to the experimentally determined ones, but they also provide an explanation for the observed biphasic trapping in MBIC11017 PSII: The fast component derives from trapping within PSII cores, while the long component derives from PSII‐Pcb supercomplexes. Despite this longer trapping time in MBIC11017 PSII, its quantum efficiency of charge separation is still high (*c*. 82%) and comparable to Chl*a‐*containing PSII (Croce & van Amerongen, 2020).

We note that the trapping time of the MBIC11017 PSII core (*c*.115 ps) is slightly longer than both isolated Chl*a* PSII cores (60–100 ps) and the fast PSII component measured in *Prochlorococcus* (97 ps). While experimental evidence for the energy level changes within the MBIC11017 PSII RC is strong (Allakhverdiev *et al*., 2011; Viola *et al*., 2022), it is possible that the driving force for the formation of the P_D1_^+^Pheo_D1_^−^ radical pair is slightly overestimated due to the overestimation of the primary donor energy, and due to the application of a 77‐mV correction for the loss of the Mn_4_CaO_5_ cluster, which may not be the precise value in Chl*d* PSII. A smaller driving force would explain the longer trapping experimentally observed in MBIC11017 PSII cores. Furthermore, investigation into the origin of the redox potential changes in both Pheo_D1_ and *Q*_A_ is required. The examination of the MBIC11017 D1 and D2 sequences does not reveal any specific changes that would explain the redox potential adjustments of either Pheo_D1_ or *Q*_A_ in Chl*d* PSII.

### Chromatic acclimation in MBIC11017

MBIC11017 is unique amongst *A. marina* strains, as it is the only strain that possesses a PBS antenna system. The phycocyanin rods, which make up the PBS, have an absorption maximum at *c*. 615 nm, which is seemingly counter‐intuitive in an organism that has adapted to FRL. However, PBS acquisition in MBIC11017 appears to be a relatively newer trait compared with FRL absorption (Ulrich *et al*., 2021), most likely reflecting adaptation to a specific spectral niche not occupied by other *A. marina* strains (Ulrich *et al*., 2024). This is exemplified by the MBIC11017 CA response in which the PBS content changes depending on the incident light quality.

As observed previously (Kashimoto *et al*., 2020) (Duxbury *et al*., 2009), RT absorption spectra show that MBIC11017 growth in WL leads to a larger PBS content relative to FRL. As evidenced by the target analyses (Fig. 5) and by the fluorescence excitation spectra of PSII in its closed state (Fig. 1b), we observe that the majority of these PBS are associated with PSII. Furthermore, the rates of EET transfer within the PBS, and from the PBS to PSII, do not change in WL‐ or FRL‐grown cells (Figs 4, 5). These data indicate that the size of each PBS does not change during the CA response (i.e. the phycocyanin rods do not become longer or shorter, and they do not bind more or less pigments), but rather only the expression levels of the PBS change, resulting in an increased PBS/PSII ratio.

MBIC11017 possesses three genes for the PBS‐membrane linker protein, CpcL (AM1_C0092, AM1_C0102 and AM1_C0203). In other species of cyanobacteria, CpcL has been shown to be an important component in a different type of rod‐shaped PBS (called CpcL‐PBS) that specifically binds to PSI due to the CpcL *N*‐terminal transmembrane domain. It is thought that the MBIC11017 PBS and the CpcL‐PBS found in many cyanobacteria are structurally similar to one another (Guo *et al*., 2024). When MBIC11017 was grown in orange light, Kashimoto *et al*. (2020) observed a large increase in the transcript level of one of these genes (AM1_C0092). We speculate that the protein encoded by AM1_C0092 specifically associates with PSII and facilitates higher levels of PSII‐associated PBS when MBIC11017 is grown in WL. It then follows that the two remaining cpcL genes encode for proteins, which are specific to PSI and PSII, although this remains to be proven.

**Fig. 7:**
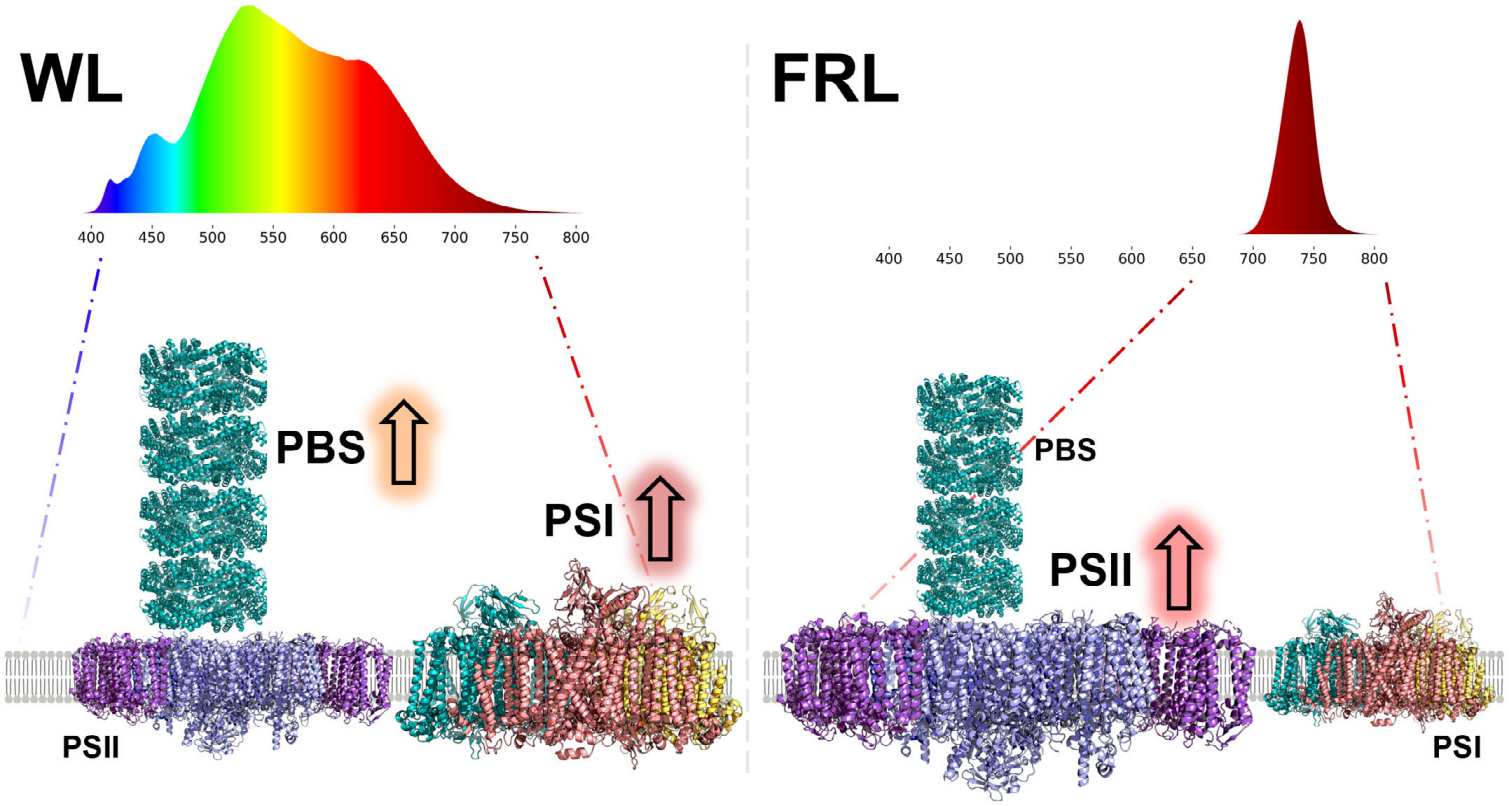
Schematic representation of the chromatic acclimation response in *Acaryochloris marina* MBIC11017. In white light (WL), MBIC11017 increases its phycobilisome (PBS) content associated with Photosystem II (PSII), as well as the relative amount of Photosystem I (PSI) (represented by the orange and red up‐arrows, respectively, in the left panel). In far‐red light (FRL), the amount of PSII increases relative to PSI (represented by the red up‐arrow in the right panel), but the amount of PBS associated with PSII decreases in abundance. The structures of PSI and PSII were taken from Hamaguchi *et al*. (2021) (PDB 7COY) and Shen *et al*. (2024) (PDB 7YMI), respectively. Additionally, the structure of the MBIC11017 phycocyanin trimer (Bar‐Zvi *et al*., 2018) (PDB 5OOK) was used to re‐create the MBIC11017 PBS, for which no structure currently exists.

Concomitant to these PBS changes, we also observe changes in photosystem stoichiometry during the CA response. When MBIC11017 is grown in WL, its PSI/PSII ratio increases to a level approximately twice that in WL. Changes in PSI/PSII ratio are a common strategy to keep the excitation balance between the two photosystems during CA in many photosynthetic organisms. However, these changes are usually the result of PSI absorbing more in the far‐red region than PSII. This is not the case in MBIC11017, in which the absorption spectra of the two photosystems are almost identical. Instead, the change in PSI/PSII ratio in MBIC11017 seems to result from the preferential association of PBS to PSII, which then requires a rebalance of the excitation between the two photosystems. This is summarised in Fig. 7.

Our TCSPC data also suggest PSII structural heterogeneity, in which PSII can exist either as a core complex or as a PSII‐Pcb supercomplex, both of which may have a PBS complex attached. The exact ratio of these different complexes changes during the CA response, with WL‐grown cells showing a larger amount of PSII core, while FRL‐grown cells possess more of the PSII‐Pcb complexes, indicating that the MBIC11017 CA response also modulates the number of PSII cores with Pcb antenna. Finally, our data also suggest that there is a population of PBS that is functionally attached to PSI (possibly via CpcL, as discussed earlier), in agreement with data from Boichenko *et al*. (2000).

### Adaptation of MBIC11017 to its unique habitat

MBIC11017 was first isolated from the ascidian, *Lissoclinum patella*, in the shallow coastal waters of Palau (Miyashita *et al*., 1996, 2003). Subsequently, many *A. marina* strains were discovered within biofilms on the underside of a variety of ascidian species (Kuhl *et al*., 2005; Ohkubo & Miyashita, 2012; Miller *et al*., 2022). However, a number of strains have also been found living epiphytically on macro‐algae (Kiang *et al*., 2022; Miller *et al*., 2022), epilithically in biofilms (Miller *et al*., 2005) or endolithically in stromatolites or crustose coralline algae (Johnson *et al*., 2022). Interestingly, the *A. marina* strains present in these latter environments typically possess a red‐shifted absorption shoulder. Ulrich *et al*. (2024) have shown that many of these strains possess photosynthetic complexes, allowing them to absorb further into the red than MBIC11017. They also demonstrated that *A. marina* strains fall into three groups: (1) long wavelength fluorescence emitters (with an emission maximum at *c*. 745 nm); (2) intermediate wavelength emitters (with an emission maximum at *c*. 730 nm); and (3) short wavelength emitters (with an emission maximum at *c*. 723 nm). MBIC11017 belongs to the short‐wavelength emitter category.

Phylogenetic analysis of these groups has shown that the most recent common ancestor of *A. marina* belonged to the intermediate‐wavelength emitter group and the short‐wavelength emitter phenotype emerged more recently in a mostly ascidian‐associated group. The longer wavelength emitter group also evolved more recently. The later emergence of the short‐wavelength emitter phenotype suggests that MBIC11017 has adapted to environments that do not experience these longer wavelengths of light. In the paragraphs that follow, we speculate on the specific environment and spectral niche that MBIC11017 occupies.

At the surface of the clear coastal waters of Palau, where the ascidian from which MBIC11017 was isolated inhabits, there is an abundance of visible light. However, this light is attenuated exponentially with depth due to the vibrational absorption bands of water, leading to distinct spectral niches in which photosynthetic organisms operate (Stomp *et al*., 2007; Holtrop *et al*., 2021). These water vibrational modes result in bluer wavelengths being attenuated weakly, while red wavelengths are attenuated more strongly. In particular, wavelengths longer than 700 nm are significantly attenuated (Pegau *et al*., 1997; Pope & Fry, 1997). Furthermore, the ascidian host also plays a role in the light spectrum available to MBIC11017. Many strains of didemnid ascidians act as hosts to the cyanobacterium *Prochloron*, which grows within the ascidian cloacal cavity. *Prochloron* spp. use Chl*a/b* as pigments but do not possess phycobilliproteins (Lewin & Withers, 1975). As a result, the light that reaches the underside of the ascidian is not only enriched in FRL but also contains a small amount of yellow‐orange light (550–650 nm) (Kuhl *et al*., 2005; Behrendt *et al*., 2012).

Consequently, ascidian‐associated *A. marina* strains occupy a spectral niche that is influenced by two factors: the light that penetrates through the ascidian and the absorption of light by water. Although the depth of water at which the *L. patella* colony was growing (from which MBIC11017 was isolated) has not been reported, Kuhl *et al*. (2005) found *A. marina* growing underneath *L. patella* at depths of 5–7 m in the shaded areas of the Heron Island outer reef slopes. The attenuation coefficient of 710‐nm light (roughly the *in vivo Q*_Y_ maximum of Chl*d*) is four times larger than at 600 nm. Therefore, at a water depth of 5 m and an equal intensity of incident light, 710‐nm light is attenuated *c*. 18 times > 600‐nm light (Pope & Fry, 1997). The two factors in combination provide a variable spectral niche for ascidian‐associated *A. marina* strains, in which the ability to harvest FRL becomes less useful at increasing depths and the ability to harvest 550‐ to 650‐nm light becomes increasingly beneficial.

MBIC11017 has evolved to take advantage of 550‐ to 650‐nm light via its PBS and its CA response. The use of a PBS by MBIC11017 makes it unique amongst *A. marina* strains, demonstrating its increased photosynthetic plasticity. Ulrich *et al*. (2021) showed that MBIC11017 has acquired its PBS genes in a horizontal gene transfer event, and the last common ancestor of *A. marina* did not possess genes encoding for PBS proteins. Phylogenetic analysis shows that MBIC11017's acquisition of PBS genes occurred after its split from the closely related *A. marina* strain, MU13, which is also a member of the short‐wavelength emitter group. Notably, MU13 is incapable of growing in monochromatic 525 or 590‐nm light, unlike MBIC11017 (Ulrich *et al*., 2021). Therefore, we postulate a possible evolutionary scenario in which an ascidian‐associated intermediate‐wavelength emitter ancestor lost its red forms (possibly to increase the quantum efficiency of charge separation) leading to the short‐wavelength emitter group. Subsequent diversification within this group and acquisition of PBS led to the evolution of MBIC11017, which represents a unique adaptation to WL by a FRL‐adapted organism.

### Conclusions

In this work, we have investigated the CA response of the Chl*d*‐containing cyanobacterium *A. marina* MBIC11017 and unveiled the excitation energy trapping dynamics of its photosystems and associated antennae using biochemical and spectroscopic techniques. Relative to their growth in FRL, MBIC11017 cells grown in WL increase their PSI/PSII ratio and increase their phycobilisome content, although the composition of the phycobilisome itself is unaffected. In both light conditions, PBS transfer their energy mostly to PSII, while a smaller portion is also directed to PSI. We show that the PSI *in vitro* trapping time is the same as the one measured *in vivo* for the cells grown in both light conditions, indicating that the PSI composition also does not change during the CA response. Moreover, we show that the PSI complex does not contain red forms, resulting in PSI and PSII possessing similar absorption spectra.

After direct excitation, energy trapping in PSII occurs within *c*. 360 ps for cells grown in WL and increases by roughly 30 ps for cells grown in FRL. Excitation energy transfer calculations using the resolved PSII structure and TRF measurements on the related divinyl Chl*a*‐containing organism *P. marinus* MIT9301 show that this trapping time is mostly determined by the PSII‐Pcb antenna arrangement, rather than by the nature of its Chl*d* reaction centre.

Combined, these data show how MBIC11017, a strain of the only species that is constitutively adapted to using FRL for photosynthesis, has adapted to growth in WL via its PBS use and CA response. This increased photosynthetic plasticity allows MBIC11017 to occupy a different spectral niche from other A. marina strains, and we speculate that this may allow the organism to live under ascidians at lower depths where FRL is lacking but yellow‐orange light is more plentiful.

## Competing interests

None declared.

## Author contributions

TJO, EE and RC conceived and designed the experiments. TJO and EE performed the experiments and the data analysis. TJO, EE and RC wrote the manuscript. RC secured funding. TJO and EE contributed equally to this work.

## Disclaimer

The New Phytologist Foundation remains neutral with regard to jurisdictional claims in maps and in any institutional affiliations.

## Supporting information


**Fig. S1** Purification of MBIC11017 trimeric Photosystem I.
**Fig. S2** SDS‐PAGE and immunoblot analysis of white light and far‐red light‐grown MBIC11017 thylakoids.
**Fig. S3** Time‐correlated single photon counting power studies of white light and far‐red light‐grown MBIC11017.
**Fig. S4** Global analysis fitting results for white light‐grown MBIC11017 cells in the open state excited at 400 nm.
**Fig. S5** Global analysis fitting results for white light‐grown MBIC11017 cells in the closed state excited at 400 nm.
**Fig. S6** Global analysis fitting results for far‐red light‐grown MBIC11017 cells in the open state excited at 400 nm.
**Fig. S7** Global analysis fitting results for far‐red light‐grown MBIC11017 cells in the closed state excited at 400 nm.
**Fig. S8** Global analysis fitting results for the isolated Photosystem I complex from white light‐grown MBIC11017.
**Fig. S9** Global analysis fitting results for white light‐grown MBIC11017 cells excited at 580 nm.
**Fig. S10** Global analysis fitting results for far‐red light‐grown MBIC11017 cells excited at 580 nm.
**Fig. S11** Global analysis fitting results for Prochlorococcus cells in the open state.
**Fig. S12** Global analysis fitting results for Prochlorococcus cells in the closed state.
**Fig. S13** Global analysis fitting results for white light‐grown MBIC11017 cells in the open state using the time‐correlated single photon counting set‐up.
**Fig. S14** Global analysis fitting results for far‐red light‐grown MBIC11017 cells in the open state using the time‐correlated single photon counting set‐up.
**Fig. S15** Global analysis fitting results for white light‐grown MBIC11017 cells in the closed state using the time‐correlated single photon counting set‐up.
**Fig. S16** Global analysis fitting results for far‐red light‐grown MBIC11017 cells in the closed state using the time‐correlated single photon counting set‐up.
**Fig. S17** Global analysis fitting results for Prochlorococcus cells in the open state using the time‐correlated single photon counting set‐up.
**Fig. S18** Global analysis fitting results for Prochlorococcus cells in the closed state using the time‐correlated single photon counting set‐up.
**Fig. S19** Target analysis fitting results for white light‐grown MBIC11017 cells excited at 400 nm.
**Fig. S20** Target analysis fitting results for white light‐grown MBIC11017 cells excited at 580 nm.
**Fig. S21** Functional Photosystem II antenna size of white light‐ and far‐red light‐grown MBIC11017 cells excited at 630 nm.
**Fig. S22** Gaussian deconvolution analysis of the isolated MBIC11017 Photosystem I absorption spectrum at 77 K.
**Fig. S23** Normalised trapping decay‐associated spectra obtained from the global analysis of the streak camera measurements on isolated MBIC11017 Photosystem I.
**Fig. S24** Global analysis of the isolated MBIC11017 Photosystem I complex with DCMU addition.
**Fig. S25** Emission spectrum of white light‐grown MBIC11017 cells upon excitation at 440 nm.
**Fig. S26** Maximum likelihood phylogeny of PsbC, prochlorophyte Chl‐binding proteins and iron‐stress response protein sequences.
**Fig. S27** Excitation energy trapping dynamics of divinyl Chl*a*‐containing Prochlorococcus marinus MIT9301 cells.
**Methods S1** Streak camera set‐up description.
**Methods S2** Global and target analysis.
**Methods S3** Excitation energy trapping simulations.
**Methods S4** Phylogenetic tree construction.
**Notes S1** Oscillator strength of phycocyanobilin vs Chl*d*.
**Notes S2** Comparison of prochlorophyte Chl‐binding proteins antenna between Prochlorococcus marinus MIT 9301 and P. marinus MIT 9313.
**Table S1** Lifetimes and amplitudes for the biexponential fits of the energy trapping simulations of MBIC11017 Photosystem II.
Please note: Wiley is not responsible for the content or functionality of any Supporting Information supplied by the authors. Any queries (other than missing material) should be directed to the *New Phytologist* Central Office.

## Data Availability

The data that support the findings of this study are available in the Supporting Information of this article. The immunoblots used to calculate the relative protein expression levels in Fig. 1(c) are shown in Fig. S2. The time‐resolved fluorescence traces measured using the streak camera set‐up and fit using the global analyses in Figs 2, 3, 4, S27 are shown in Figs S4–S12. The fits of the target analysis presented in Fig. 5 are shown in Figs S19 and S20. The time‐resolved fluorescence traces measured using the TCSPC set‐up and fit using the global analyses in Figs 6(a,b), S27 are shown in Figs S13–S18. Additionally the source data can be found at doi:10.5281/zenodo.15308510.
