# Supplementary material for: Acclimation to white light in a far‐red light specialist: insights from Acaryochloris marina MBIC11017
Source: New Phytol. 2025 May 5;247(1):128–43. doi: 10.1111/nph.70188 (PMC12138183; doi:10.1111/nph.70188)
Supplement: Supplementary file 1 — Fig. S1 Purification of MBIC11017 trimeric Photosystem I. Fig. S2 SDS‐PAGE and immunoblot analysis of white light and far‐red light‐grown MBIC11017 thylakoids. Fig. S3 Time‐correlated single photon counting power studies of white light and far‐red light‐grown MBIC11017. Fig. S4 Global analysis fitting results for white light‐grown MBIC11017 cells in the open state excited at 400 nm. Fig. S5 Global analysis fitting results for white light‐grown MBIC11017 cells in the closed state excited at 400 nm. Fig. S6 Global analysis fitting results for far‐red light‐grown MBIC11017 cells in the open state excited at 400 nm. Fig. S7 Global analysis fitting results for far‐red light‐grown MBIC11017 cells in the closed state excited at 400 nm. Fig. S8 Global analysis fitting results for the isolated Photosystem I complex from white light‐grown MBIC11017. Fig. S9 Global analysis fitting results for white light‐grown MBIC11017 cells excited at 580 nm. Fig. S10 Global analysis fitting results for far‐red light‐grown MBIC11017 cells excited at 580 nm. Fig. S11 Global analysis fitting results for Prochlorococcus cells in the open state. Fig. S12 Global analysis fitting results for Prochlorococcus cells in the closed state. Fig. S13 Global analysis fitting results for white light‐grown MBIC11017 cells in the open state using the time‐correlated single photon counting set‐up. Fig. S14 Global analysis fitting results for far‐red light‐grown MBIC11017 cells in the open state using the time‐correlated single photon counting set‐up. Fig. S15 Global analysis fitting results for white light‐grown MBIC11017 cells in the closed state using the time‐correlated single photon counting set‐up. Fig. S16 Global analysis fitting results for far‐red light‐grown MBIC11017 cells in the closed state using the time‐correlated single photon counting set‐up. Fig. S17 Global analysis fitting results for Prochlorococcus cells in the open state using the time‐correlated single photon counting set‐up. Fig. S18 Gl [file NPH-247-128-s001.docx]

## *New Phytologist* Supporting Information

Article title: Acclimation to White Light in a Far-Red Light Specialist: Insights from *Acaryochloris marina* MBIC11017

Authors: Thomas J. Oliver, Eduard Elias, and Roberta Croce

Article acceptance date: 2 April 2025

The following Supporting Information is available for this article:

**Fig. S1** Purification of MBIC11017 Trimeric PSI.

**Fig. S2** SDS PAGE and Immunoblot analysis of WL and FRL grown MBIC11017 thylakoids.

**Fig. S3** TCSPC power studies of WL and FRL grown MBIC11017.

**Fig. S4** Global analysis fitting results for WL grown MBIC11017 cells in the open state excited at 400 nm.

**Fig. S5** Global analysis fitting results for WL grown MBIC11017 cells in the closed state excited at 400 nm.

**Fig. S6** Global analysis fitting results for FRL grown MBIC11017 cells in the open state excited at 400 nm.

**Fig. S7** Global analysis fitting results for FRL grown MBIC11017 cells in the closed state excited at 400 nm.

**Fig. S8** Global analysis fitting results for the isolated PSI complex from WL grown MBIC11017.

**Fig. S9** Global analysis fitting results for WL grown MBIC11017 cells excited at 580 nm.

**Fig. S10** Global analysis fitting results for FRL grown MBIC11017 cells excited at 580 nm.

**Fig. S11** Global analysis fitting results for *Prochlorococcus* cells in the open state.

**Fig. S12** Global analysis fitting results for *Prochlorococcus* cells in the closed state.

**Fig. S13** Global analysis fitting results for WL grown MBIC11017 cells in the open state using the TCSPC setup.

**Fig. S14** Global analysis fitting results for FRL grown MBIC11017 cells in the open state using TCSPC setup.

**Fig. S15** Global analysis fitting results for WL grown MBIC11017 cells in the closed state using TCSPC setup.

**Fig. S16** Global analysis fitting results for FRL grown MBIC11017 cells in the closed state using TCSPC setup.

**Fig. S17** Global analysis fitting results for *Prochlorococcus* cells in the open state using TCSPC setup.

**Fig. S18** Global analysis fitting results for *Prochlorococcus* cells in the closed state using TCSPC setup.

**Fig. S19** Target analysis fitting results for WL grown MBIC11017 cells excited at 400 nm.

**Fig. S20** Target analysis fitting results for WL grown MBIC11017 cells excited at 580 nm.

**Fig. S21** Functional PSII antenna size of WL and FRL grown MBIC11017 cells excited at 630 nm.

**Fig. S22** Gaussian deconvolution analysis of the isolated MBIC11017 PSI absorption spectrum at 77 K.

**Fig. S23** Normalized trapping DAS obtained from the global analysis of the streak camera measurements on isolated MBIC11017 PSI.

**Fig. S24** Global analysis of the isolated MBIC11017 PSI complex with DCMU addition.

**Fig. S25** Emission spectrum of WL grown MBIC11017 cells upon excitation at 440 nm.

**Fig. S26** Maximum likelihood phylogeny of PsbC, Pcb and IsiA sequences.

**Fig. S27** Excitation energy trapping dynamics of divinyl-Chl *a*-containing *Prochlorococcus marinus* MIT9301 cells.

**Table S1** Lifetimes and amplitudes for the bi-exponential fits of the energy trapping simulations of MBIC11017 PSII.

**Methods S1** Streak camera setup description.

**Methods S2** Global and target Analysis.

**Methods S3** Excitation energy trapping simulations.

**Methods S4** Phylogenetic tree construction.

**Notes S1** The oscillator strength of phycocyanobilin vs chlorophyll *d*.

**Notes S2** Comparison of Pcb antenna between *P. marinus* MIT 9301 with *P. marinus* MIT 9313.

**Fig. S1 Purification of MBIC11017 Trimeric PSI. (A)** Sucrose density gradient of WL MBIC11017 solubilised thylakoids. The lowest major band contained trimeric PSI. **(B)** Sucrose density gradient of cleaned MBIC11017 trimeric PSI. **(C)** 12% tricine SDS PAGE gel of MBIC11017 WL Thylakoids (Lane 1), trimeric PSI (lane 2) and cleaned trimeric (PSI). Samples were loaded at a Chl concentration of 0.5 µg **(D)** Immunoblot using PsaB and CP47 antibodies to show minimal contamination of PSII in the cleaned trimeric PSI.


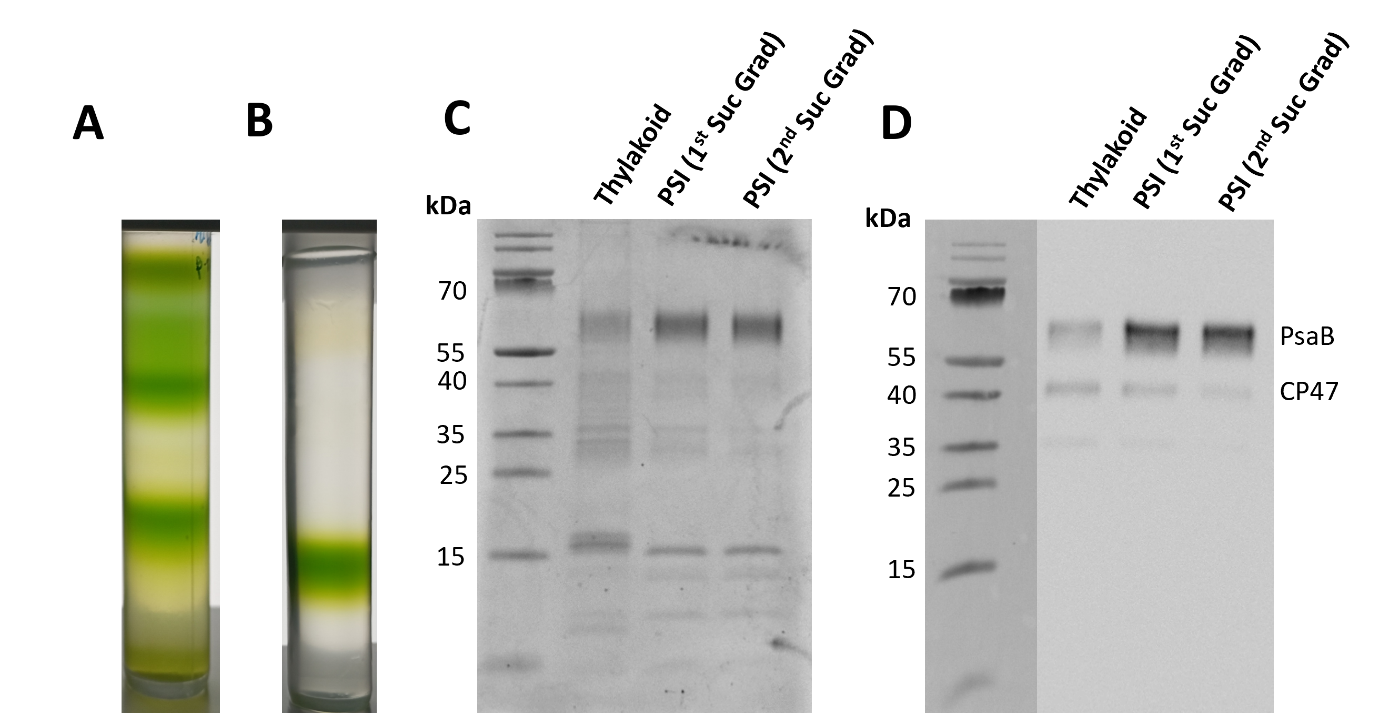


**Fig. S2 SDS PAGE and Immunoblot analysis of WL and FRL grown MBIC11017 thylakoids. (A)** 12% tricine SDS PAGE gel of WL and FRL grown MBIC11017 thylakoid at concentrations of 0.25, 0.5 and 1 µg of Chl. Two biological replicates were loaded for each sample. **(B)** Immunoblot of an SDS-PAGE gel obtained in the same manner as **Fig. S2A** using PsaB and CP47 antibodies. **(C)** Immunoblot of an SDS-PAGE gel obtained in the same manner as **Fig. S2A** using PsaB and IsiA antibodies.

**
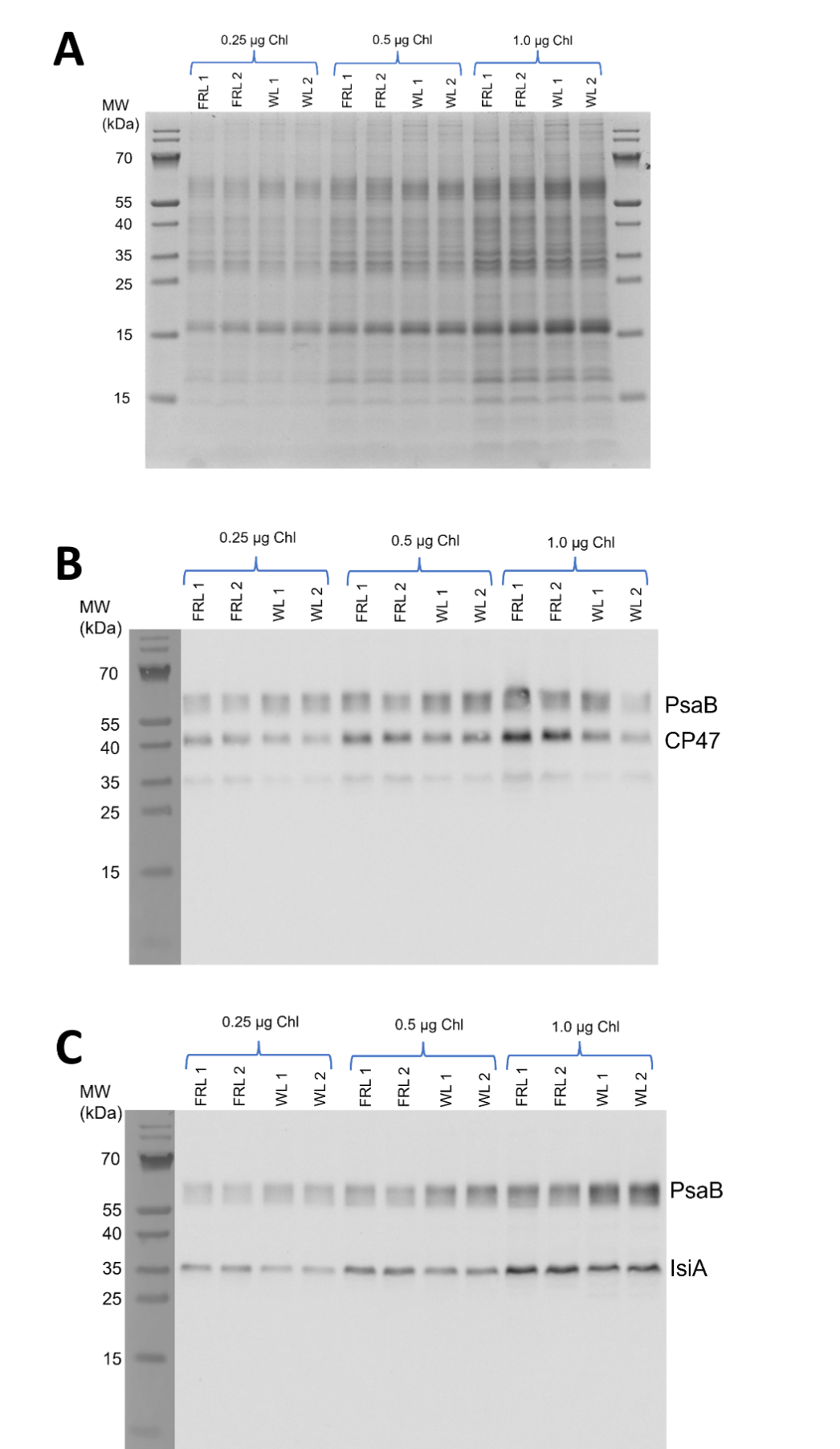
**

**Fig. S3 TCSPC Power studies of WL (A) and FRL (B) grown MBIC11017 cells after excitation at 440 nm.** All traces were recorded until reaching 3000 counts at their maximum. The traces are independent of excitation power when using 0.5 µW or less, which was taken into account in order to obtain DAS with PSII in its open state (**Fig. 6A** & **B**).


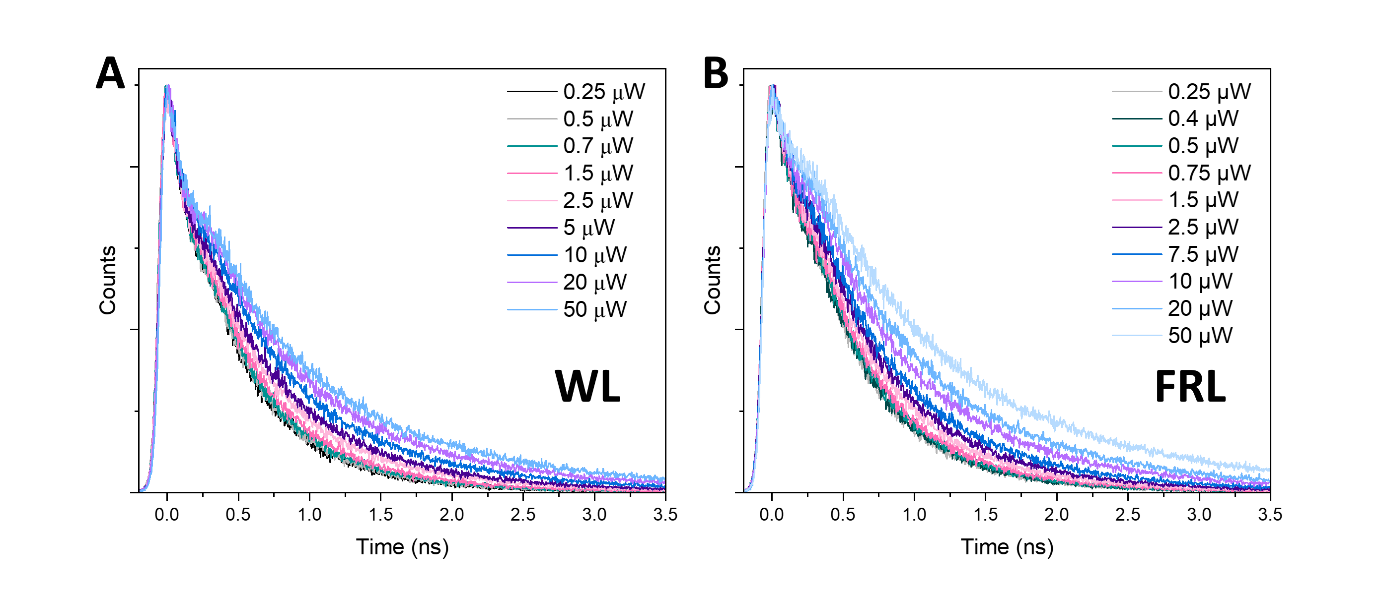


**Fig. S4 Global analysis fitting results for WL grown MBIC11017 cells in the open state excited at 400 nm.** Data was measured with a streak camera setup using 400 nm excitation with PSII in the open state. Global analysis of this data is shown in **Fig. 2A**.


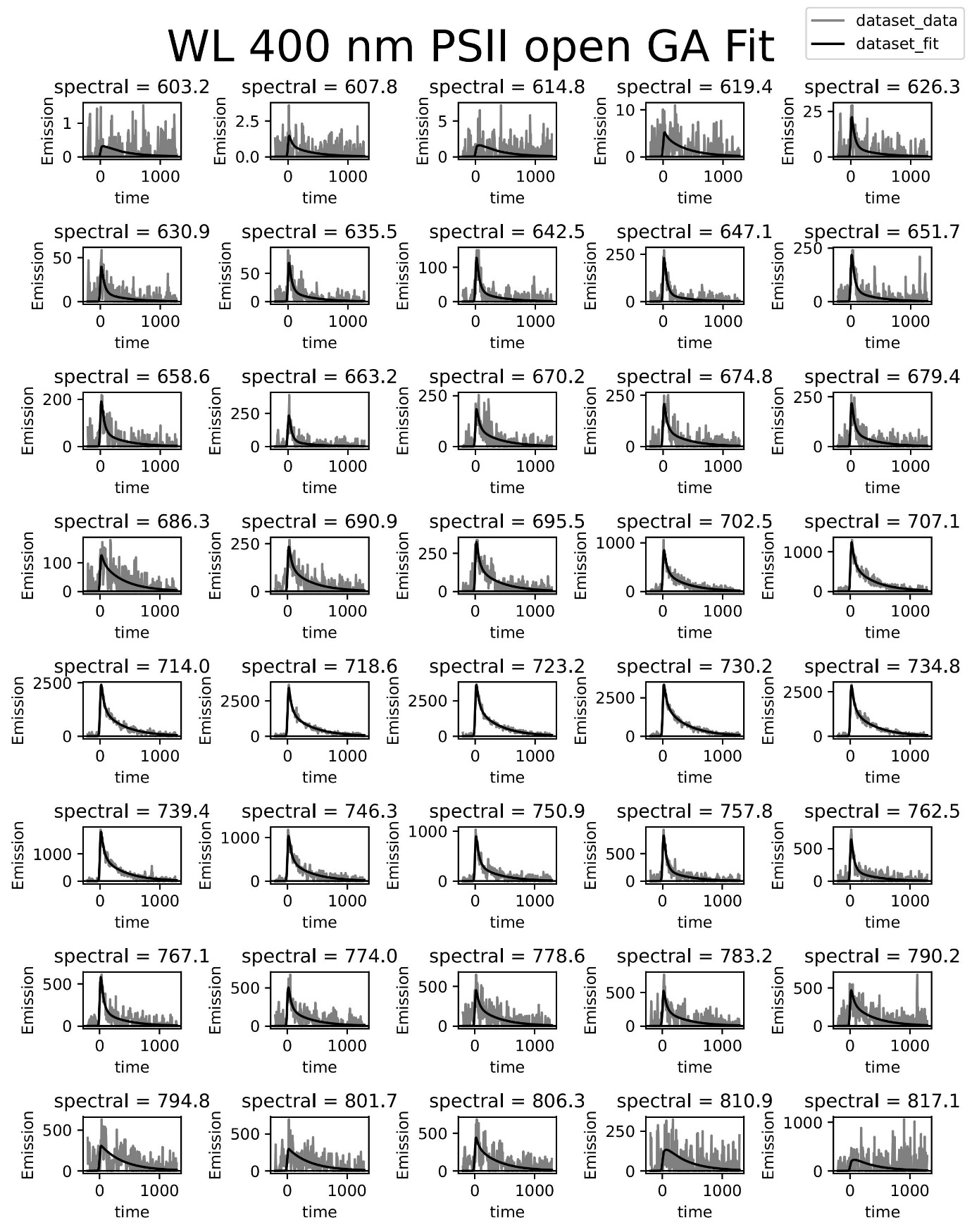


**Fig. S5 Global analysis fitting results for WL grown MBIC11017 cells in the closed state excited at 400 nm.** Data was measured with a streak camera setup using 400 nm excitation with PSII in the closed state. Global analysis of this data is shown in **Fig. 2B**.


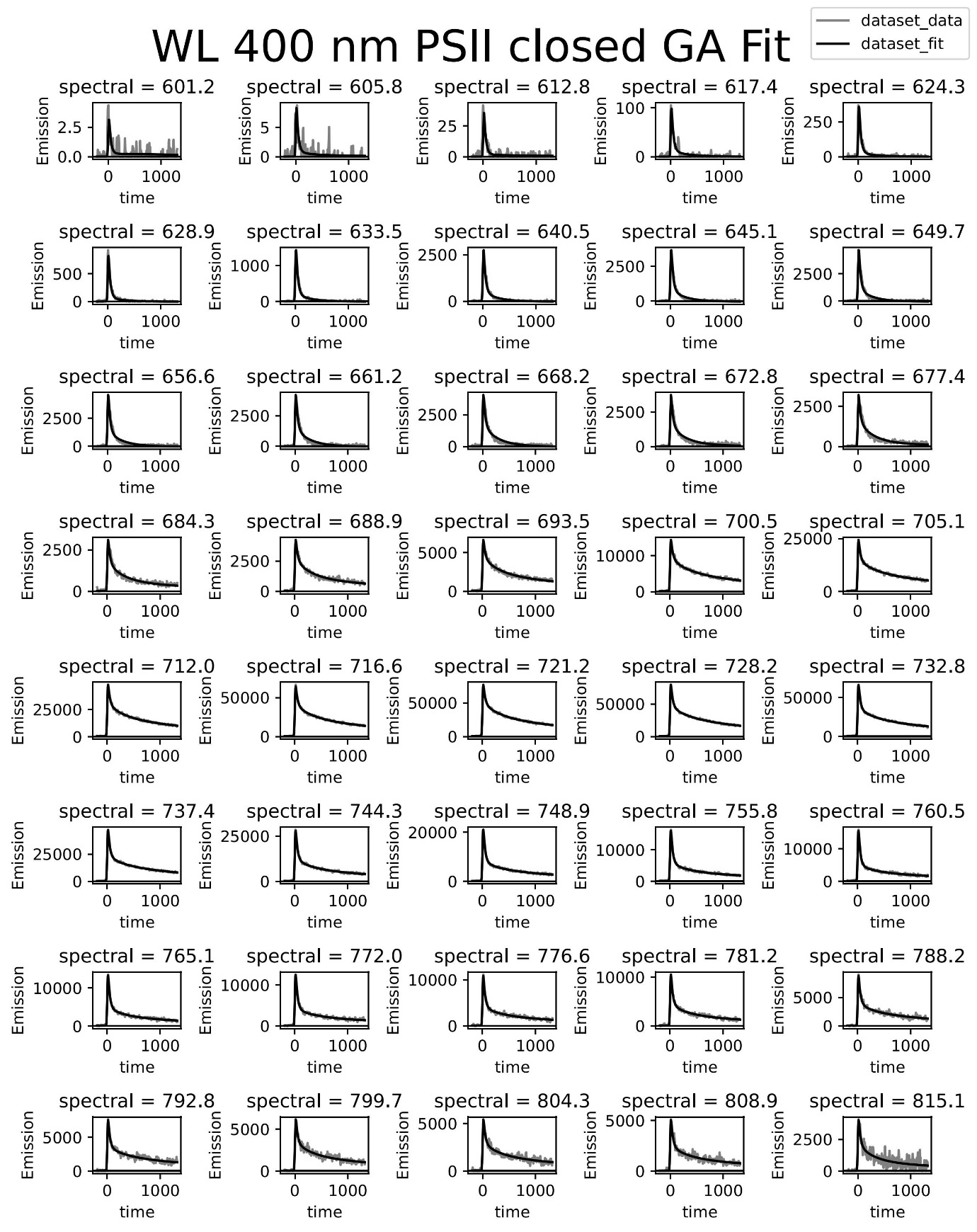


**Fig. S6 Global analysis fitting results for FRL grown MBIC11017 cells in the open state excited at 400 nm.** Data was measured with a streak camera setup using 400 nm excitation with PSII in the open state. Global analysis of this data is shown in **Fig. 2C**.


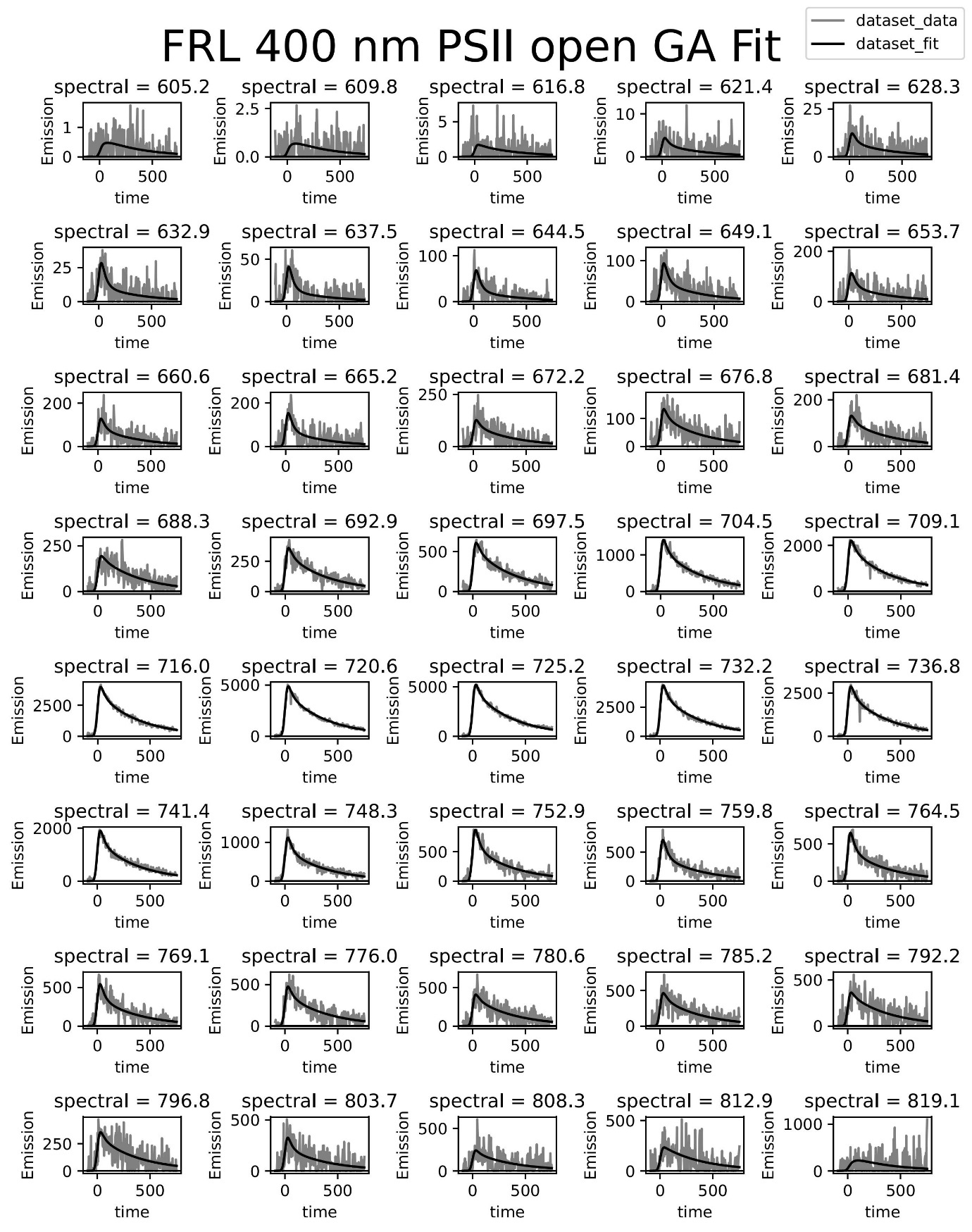


**Fig. S7 Global analysis fitting results for FRL grown MBIC11017 cells in the closed state excited at 400 nm.** Data was measured with a streak camera setup using 400 nm excitation with PSII in the closed state. Global analysis of this data is shown in **Fig. 2D**.


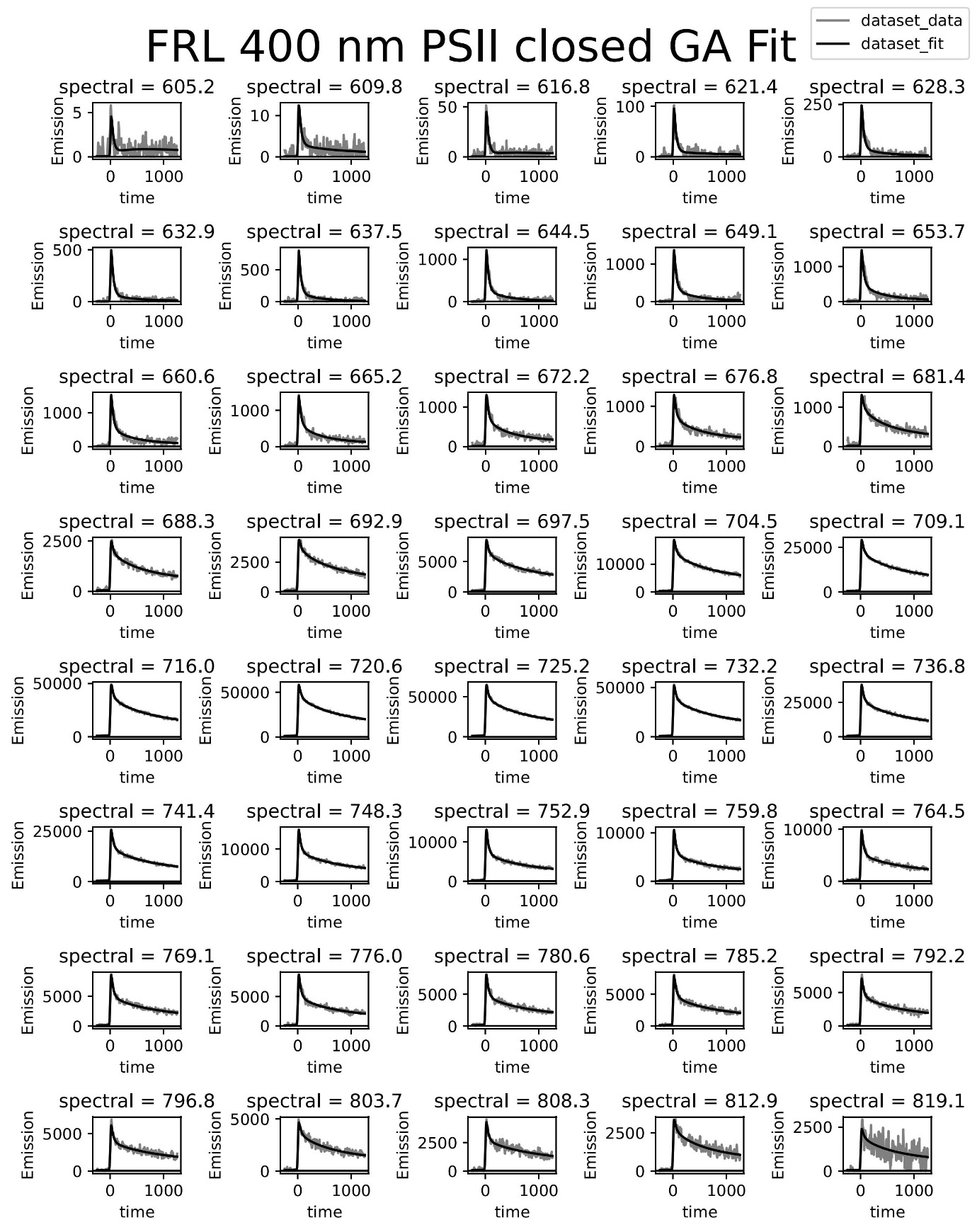


**Fig. S8 Global analysis fitting results for the isolated PSI complex from WL grown MBIC11017.** Data was measured with a streak camera setup using 400 nm excitation. Global analysis of this data is shown in **Fig. 3C**.


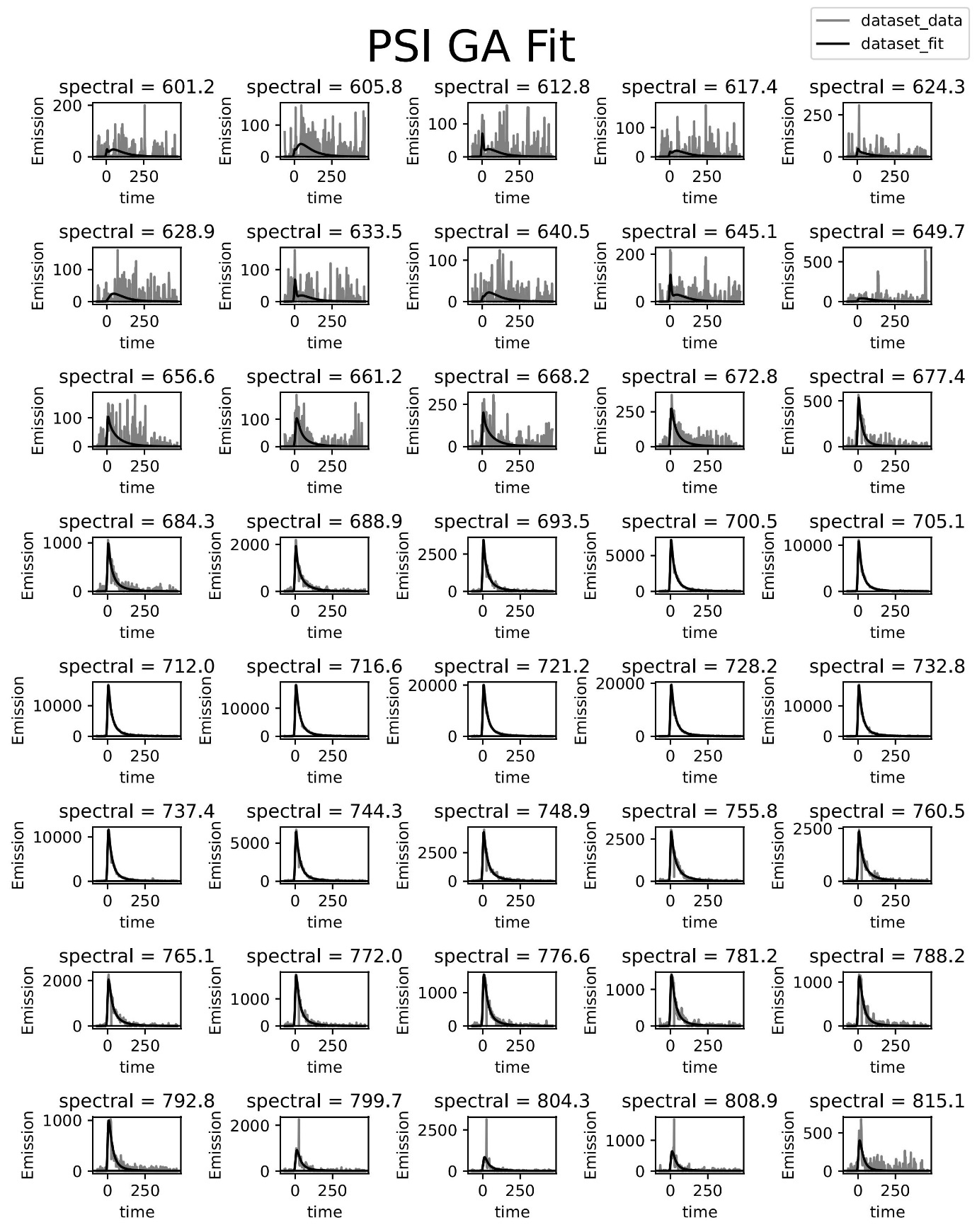


**Fig. S9 Global analysis fitting results for WL grown MBIC11017 cells excited at 580 nm.** Data was measured with a streak camera setup using 580 nm excitation with PSII in the open state. Global analysis of this data is shown in **Fig. 4A**.


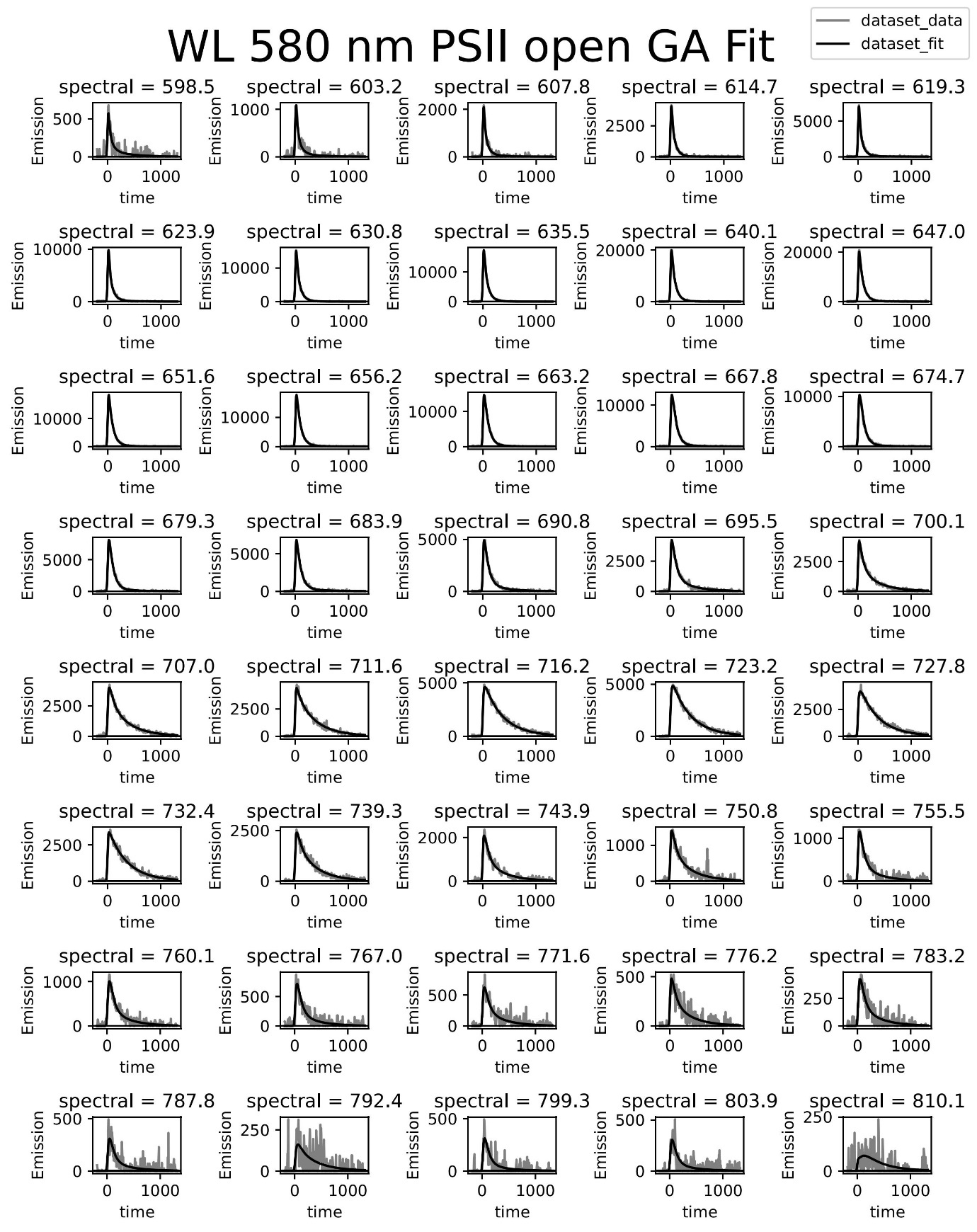


**Fig. S10 Global analysis fitting results for FRL grown MBIC11017 cells excited at 580 nm.** Data was measured with a streak camera setup using 580 nm excitation with PSII in the open state. Global analysis of this data is shown in **Fig. 4B**.


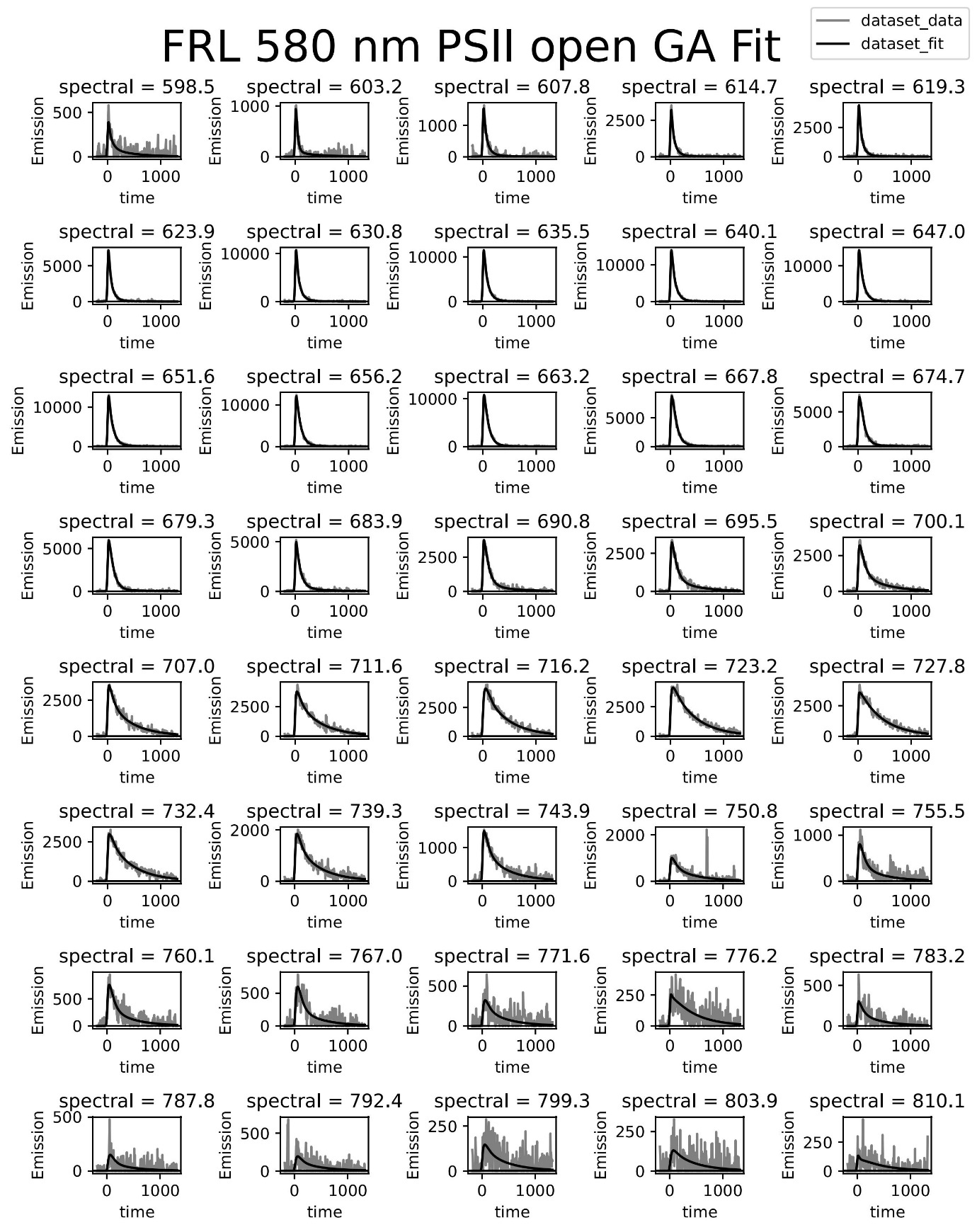


**Fig. S11 Global analysis fitting results for *Prochlorococcus* cells in the open state.** Data was measured with a streak camera setup using 400 nm excitation with PSII in the open state. Global analysis of this data is shown in **Fig. S27A**.


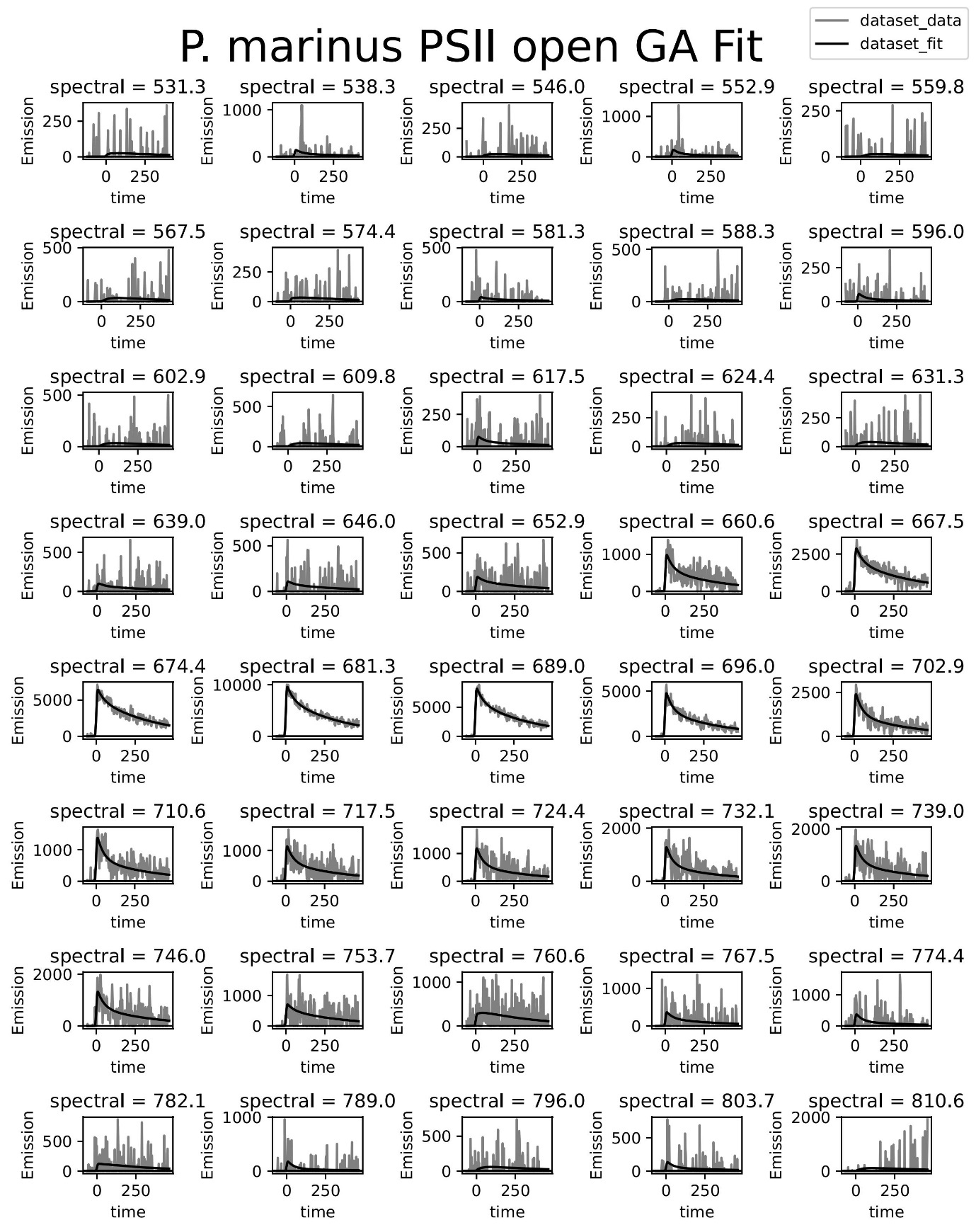


**Fig. S12 Global analysis fitting results for *Prochlorococcus* cells in the closed state.** Data was measured with a streak camera setup using 400 nm excitation with PSII in the closed state. Global analysis of this data is shown in **Fig. S27B**.


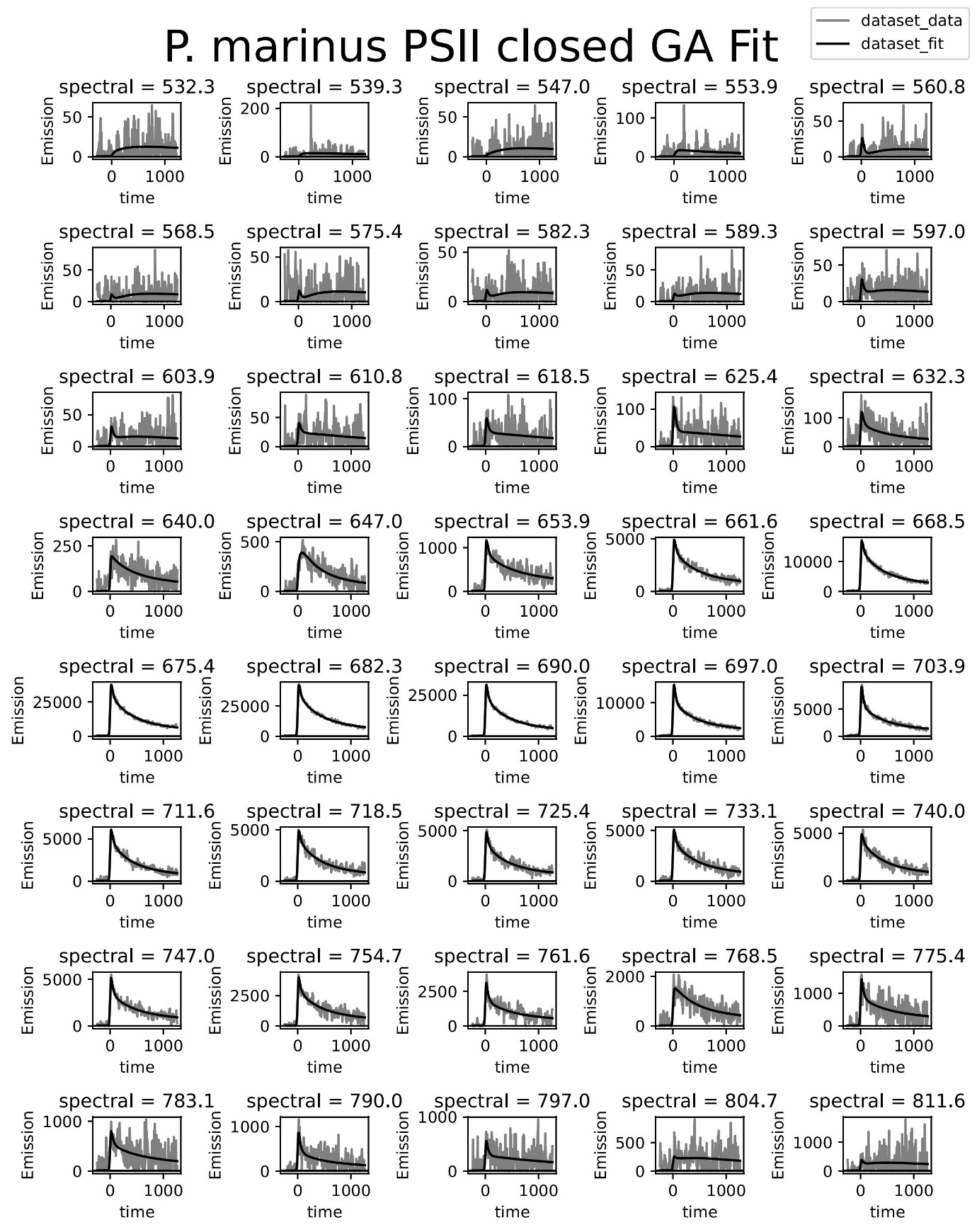


**Fig. S13 Global analysis fitting results for WL grown MBIC11017 cells in the open state.** Data was measured with a TCSPC setup using 440 nm excitation with PSII in the open state. Global analysis of this data is shown in **Fig. 6A**.

**
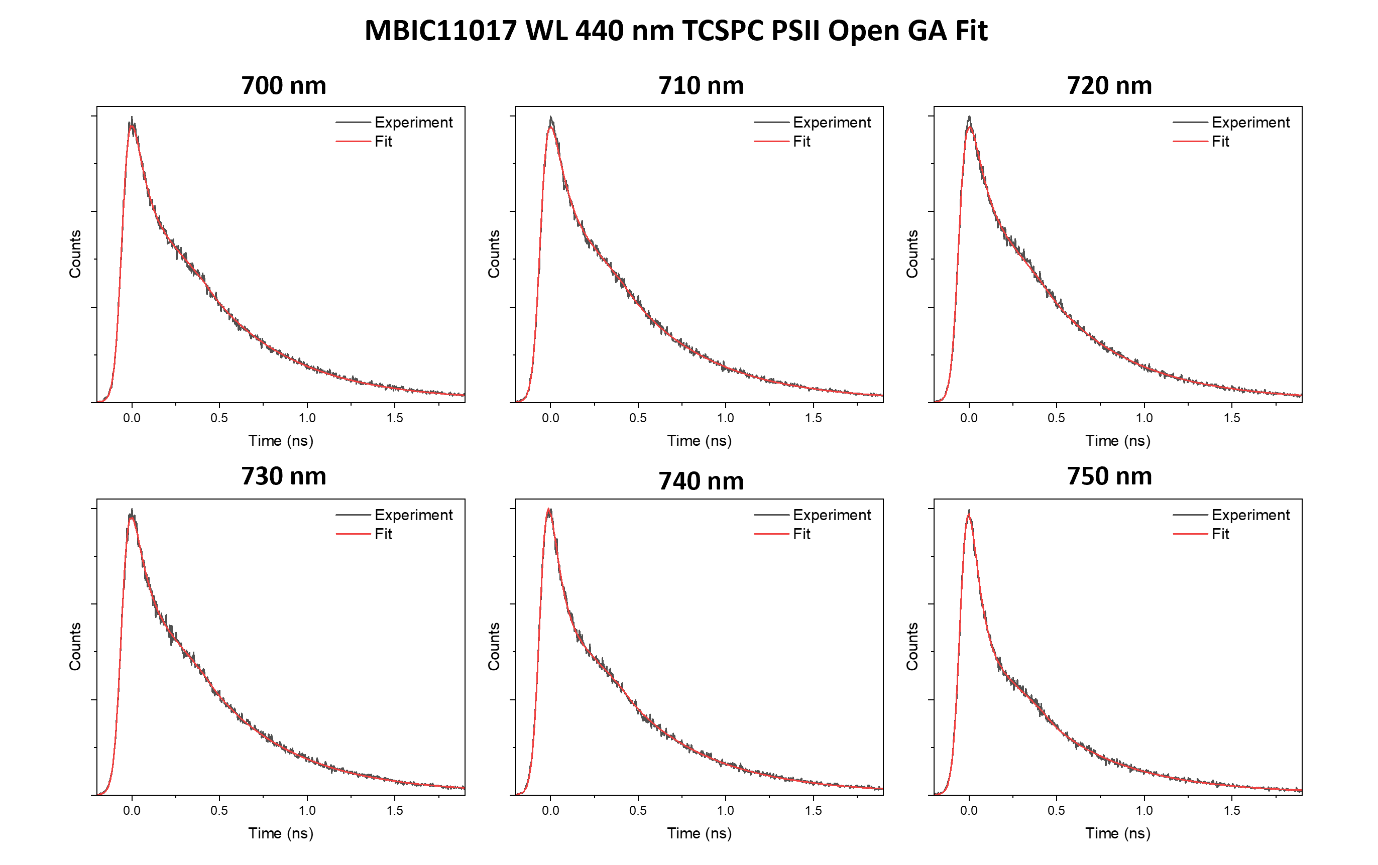
**

**Fig. S14 Global analysis fitting results for FRL grown MBIC11017 cells in the open state.** Data was measured with a TCSPC setup using 440 nm excitation with PSII in the open state. Global analysis of this data is shown in **Fig. 6B**.

**
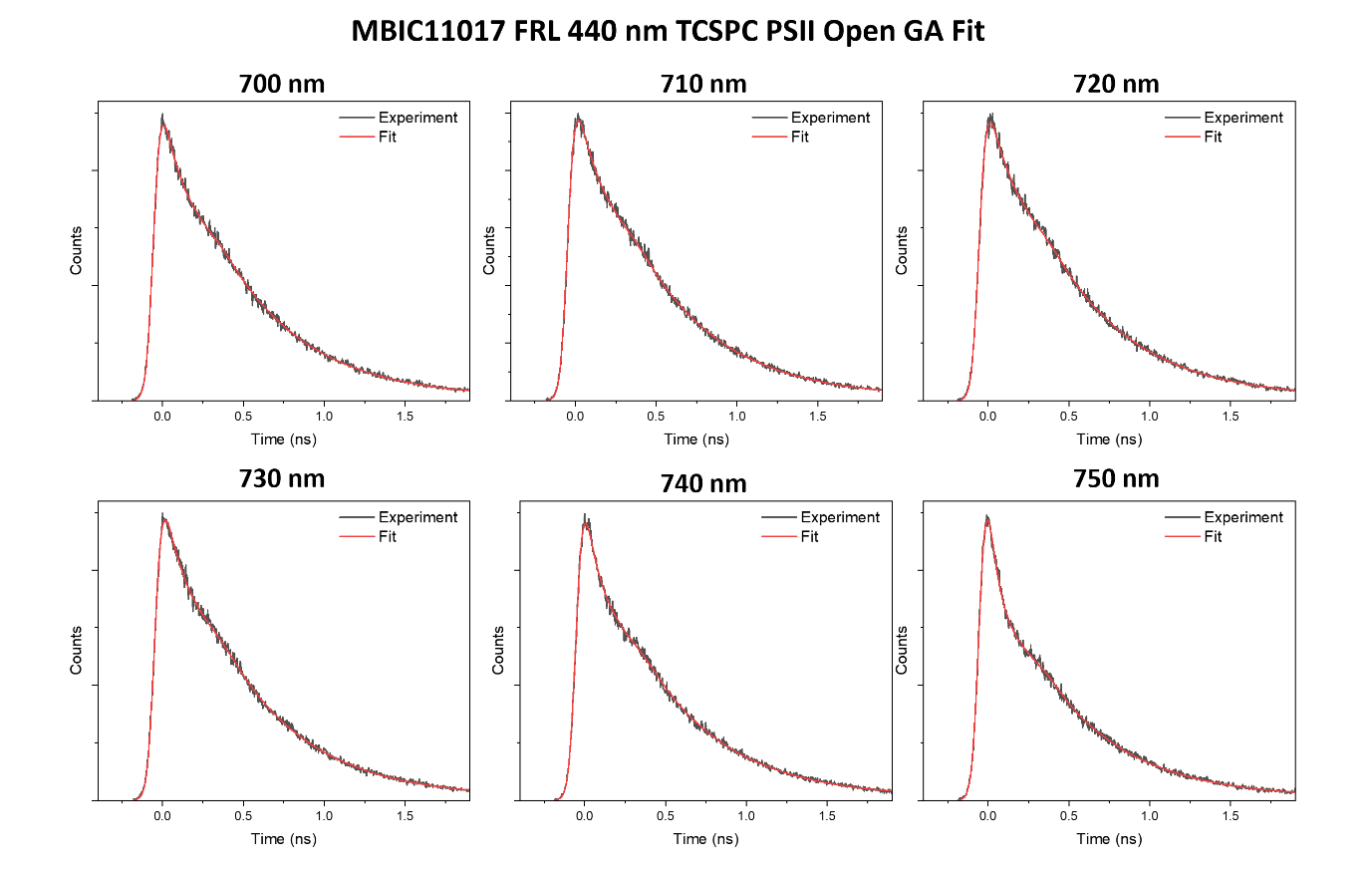
**

**Fig. S15 Global analysis fitting results for WL grown MBIC11017 cells in the closed state.** Data was measured with a TCSPC setup using 440 nm excitation with PSII in the closed state.

**
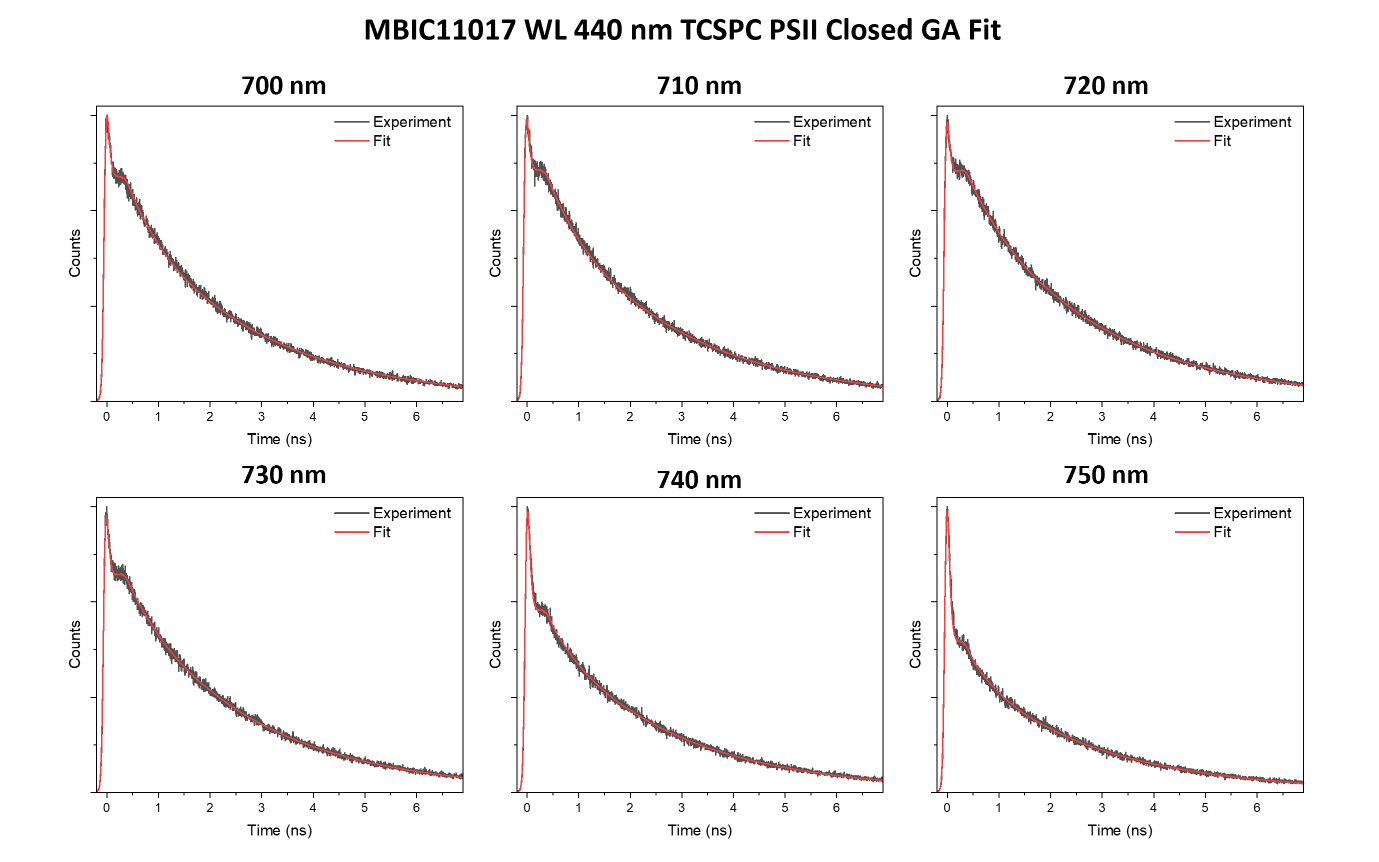
**

**Fig. S16 Global analysis fitting results for FRL grown MBIC11017 cells in the closed state.** Data was measured with a TCSPC setup using 440 nm excitation with PSII in the closed state.

**
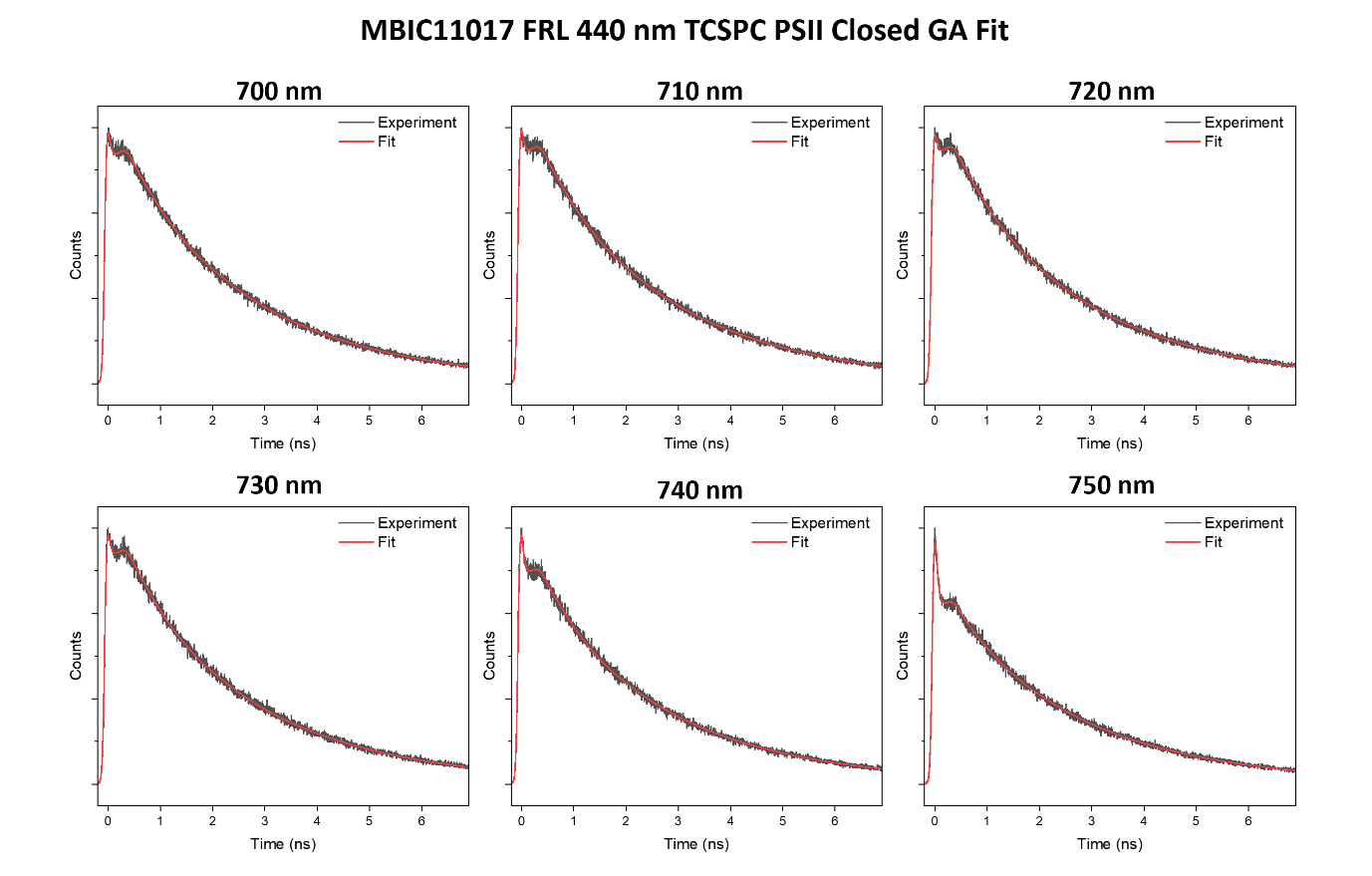
**

**Fig. S17 Global analysis fitting results for *Prochlorococcus* cells in the open state.** Data was measured with a TCSPC setup using 440 nm excitation with PSII in the open state. Global analysis of this data is shown in **Fig. S27C**.


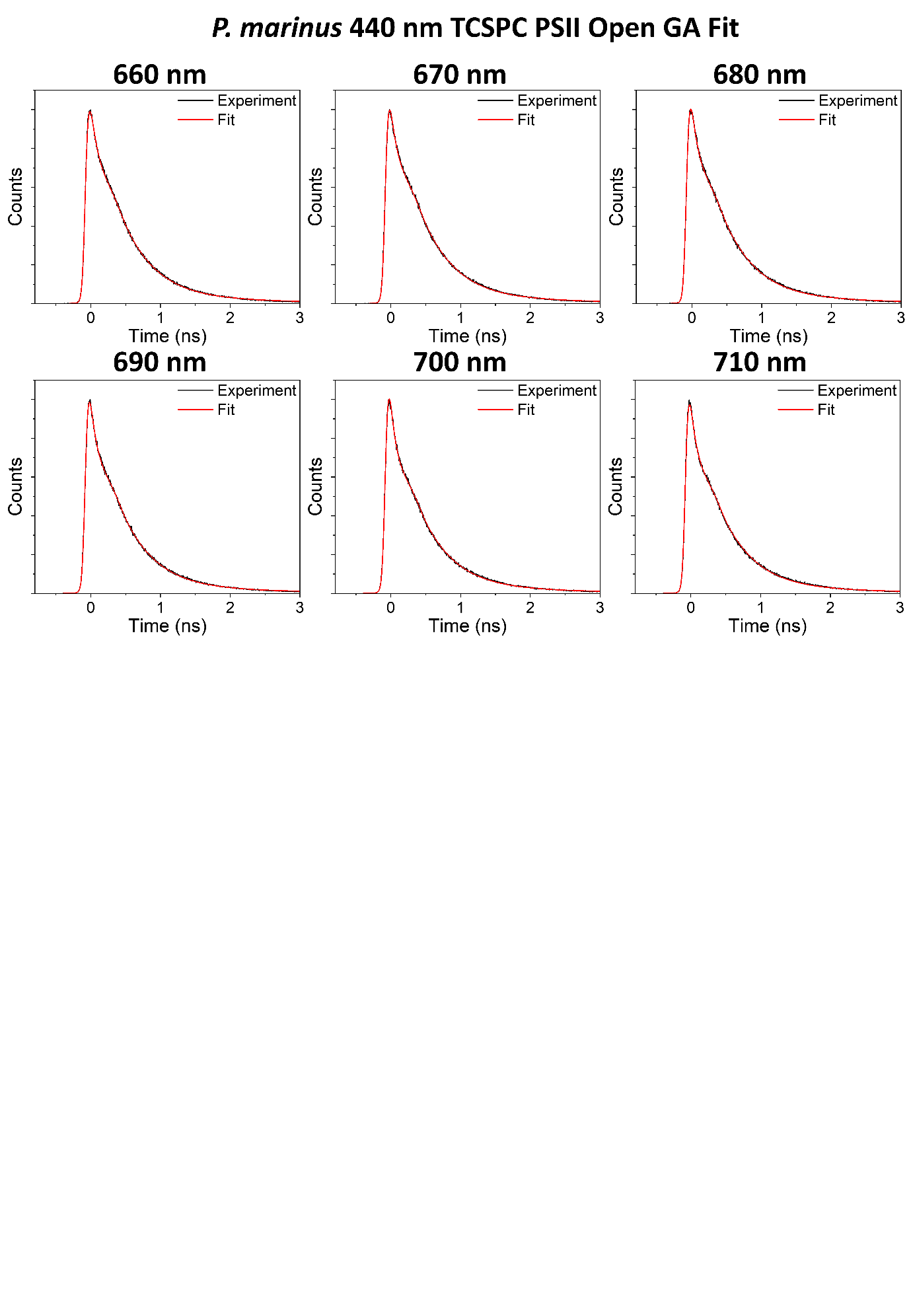


**Fig. S18 Global analysis fitting results for *Prochlorococcus* cells in the closed state.** Data was measured with a TCSPC setup using 440 nm excitation with PSII in the closed state. Global analysis of this data is shown in **Fig. S27D**.


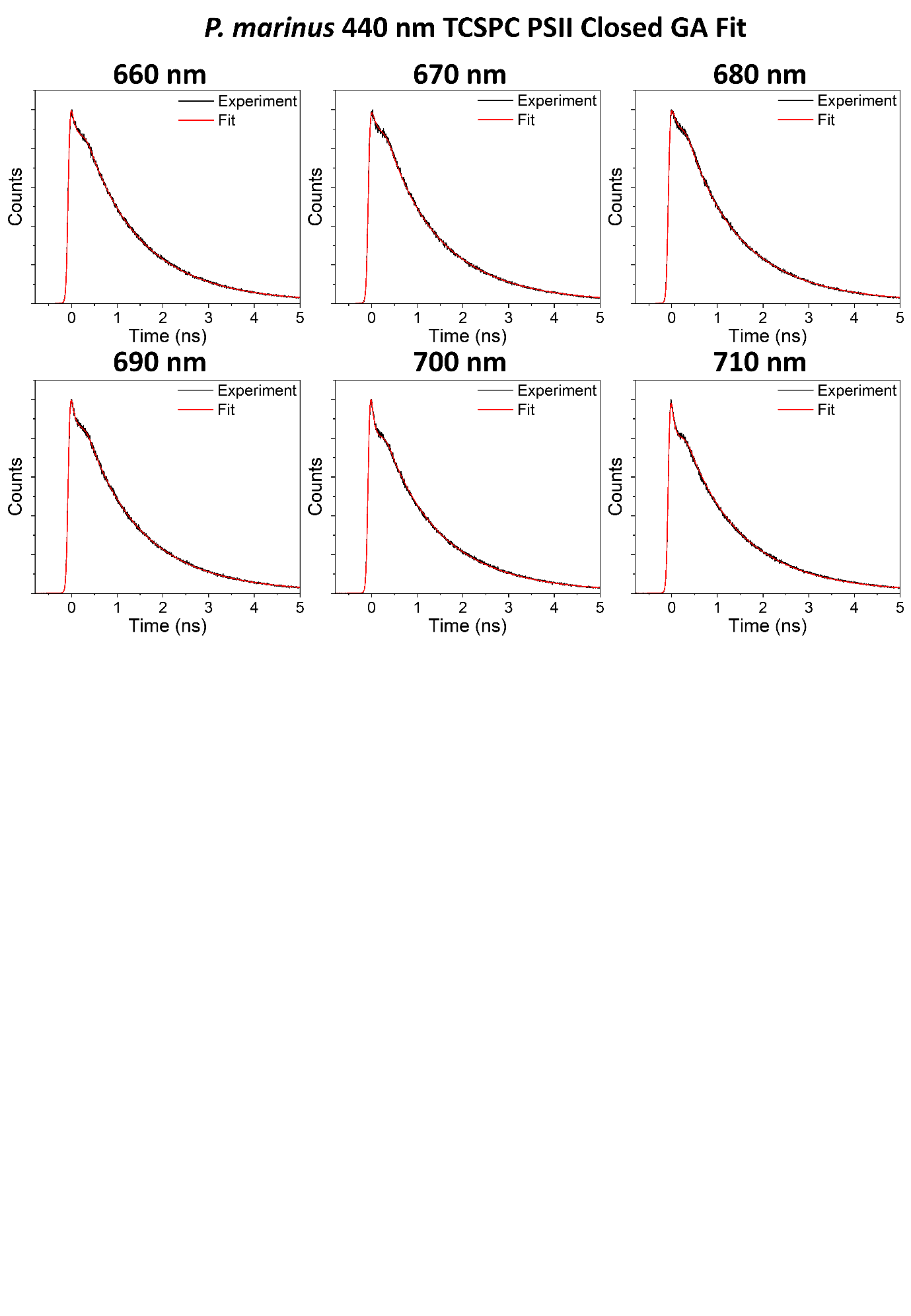


**Fig. S19 Target analysis fitting results for WL grown MBIC11017 cells excited at 400 nm.** Data was measured with a streak camera setup using 400 nm excitation with PSII in the open state. The kinetic scheme used for this target analysis is shown in **Fig. 5A** and the normalized species associated spectra obtained with these fits are shown in **Fig. 5B.**


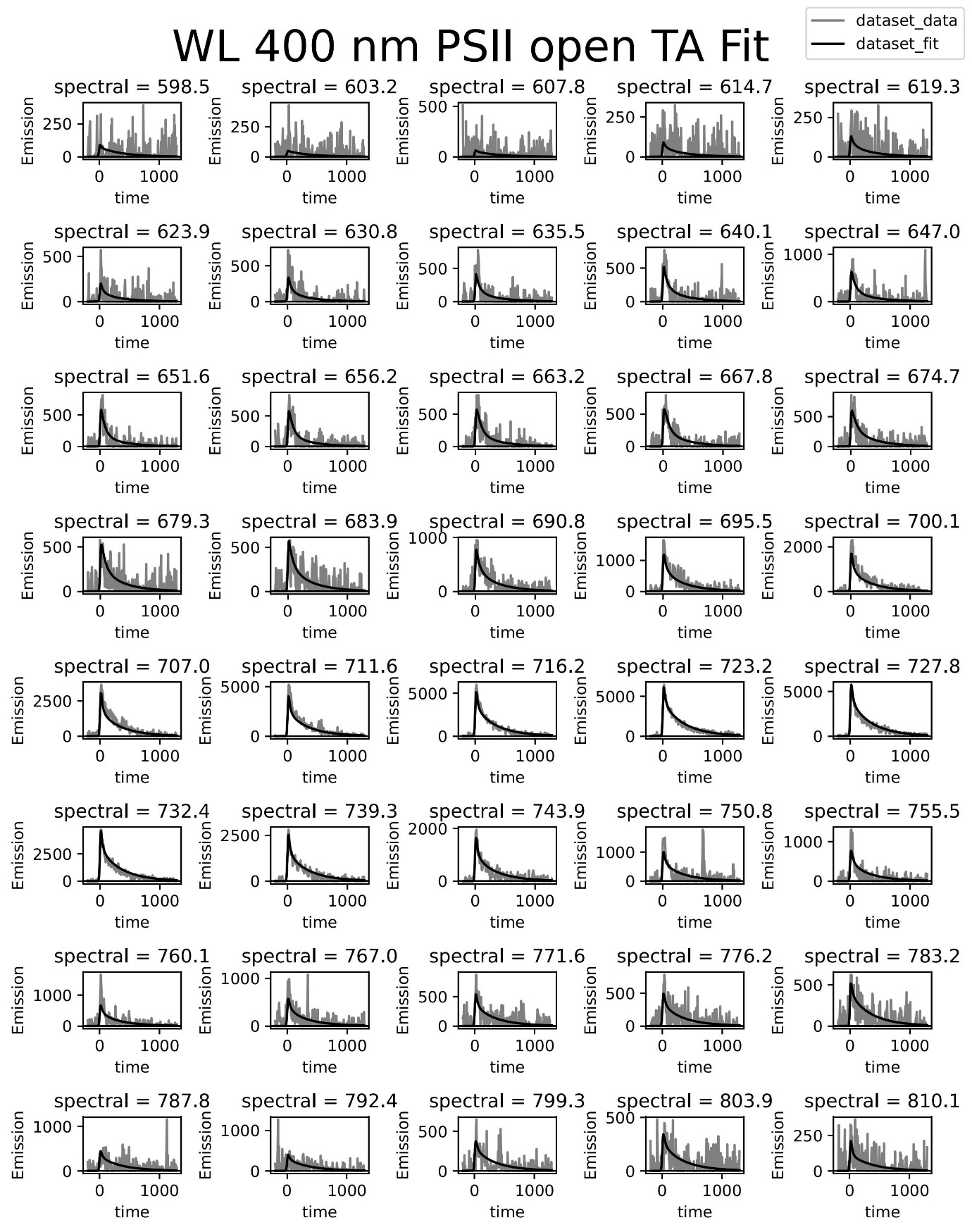


**Fig. S20 Target analysis fitting results for WL grown MBIC11017 cells excited at 580 nm.** Data was measured with a streak camera setup using 580 nm excitation with PSII in the open state. The kinetic scheme used for this target analysis is shown in **Fig. 5A** and the normalized species associated spectra obtained with these fits are shown in **Fig. 5B.**


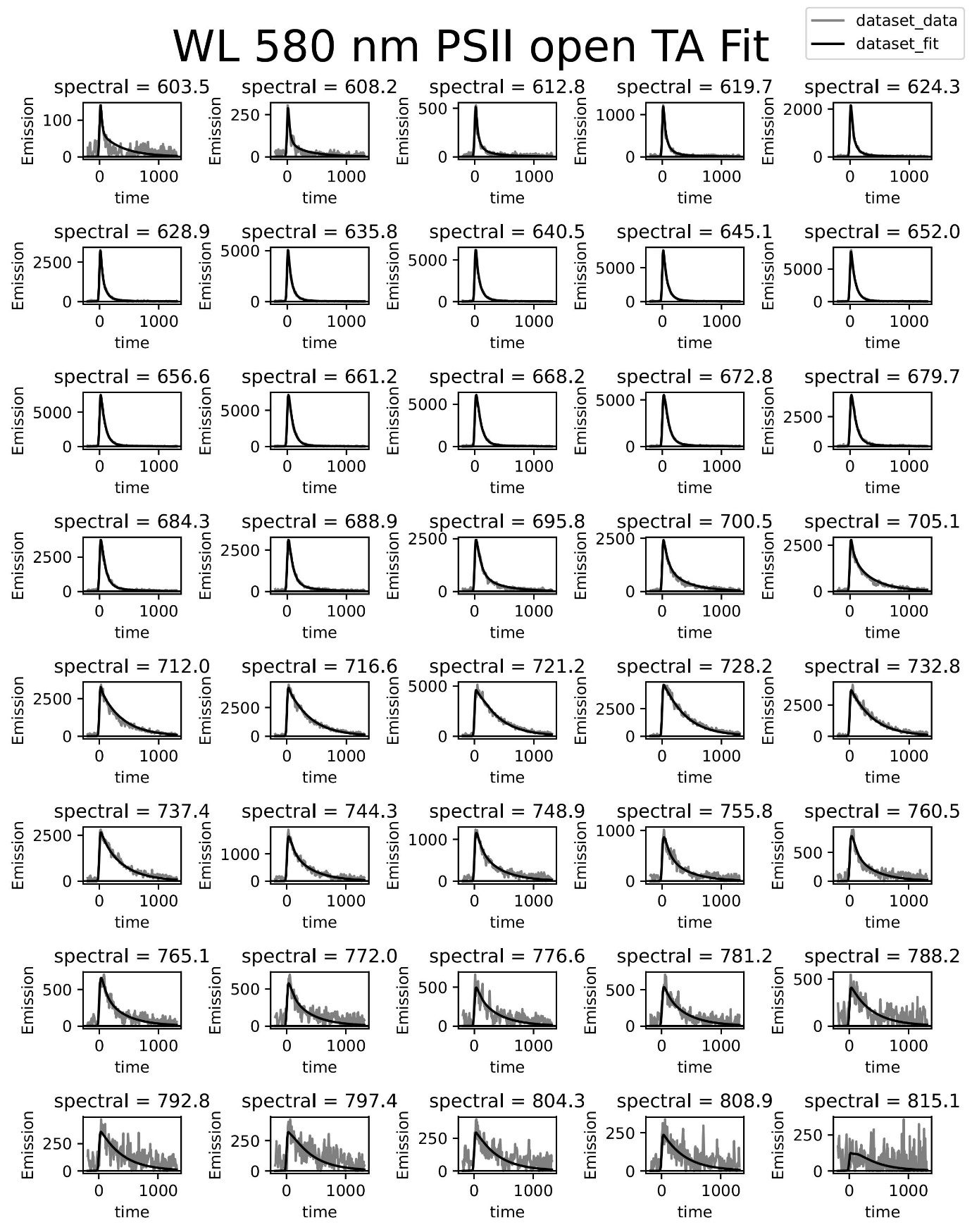


**Fig. S21 Functional PSII antenna of WL and FRL grown MBIC11017 cells when exciting at 630 nm.** A 630 nm LED was used to preferentially excite the MBIC11017 PBS and PSII fluorescence was measured using a JTS spectrophotometer. The 630 nm LED should not only excite the PBS but also the Q_X_ band of Chl *d*, therefore also exciting those PSII that do not have PBS attached. As such, the antenna size measured using this excitation wavelength should be considered the average antenna size of all PSII (given that MBIC11017 PSII cores may have a PBS and/or Pcb attached). After 630 nm excitation, the slope of the ETR as a function of the actinic light intensity is ~10% larger for WL grown cells. Considering that FRL grown cells possess a larger Chl d antenna size (**Fig. 1D**), the result here qualitatively suggests that the WL grown cells have a larger PBS associated PSII antenna size than FRL grown cells, although this method does not allow us to be quantitative.


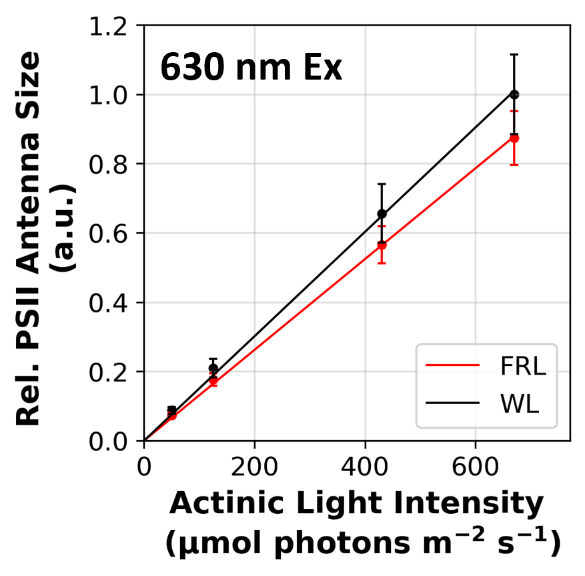


**Fig. S22 Gaussian deconvolution analysis of the isolated MBIC11017 PSI absorption spectrum at 77 K. (A)** Gaussian deconvolution of the PSI 77 K absorption spectrum in which the FWHM of all the Gaussian were constrained to be the same. **(B)** The predicted emission spectrum of the isolated PSI complex, as determined from the Boltzmann equation, taking the energy of each state as the central position of the corresponding Gaussian, and its relative area as its relative abundance. A Stokes shift of 100 cm^-1^ was applied to each Gaussian. **(C)** The same as in **B**, but excluding the two lowest energy Gaussians from the analysis.

**
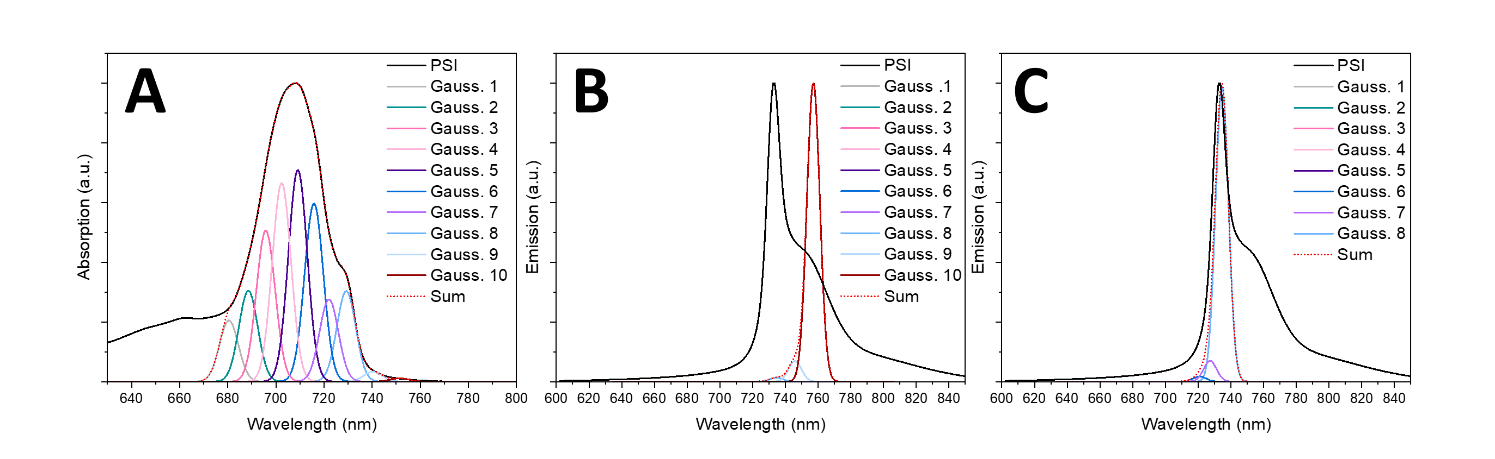
**

**Fig. S23 Normalized trapping DAS obtained from the global analysis of the streak camera measurements on isolated MBIC11017 PSI.**

**Fig. S24 Global analysis of the isolated MBIC11017 PSI complex with the addition of 100 µM of DCMU.** The values denoted after ± are standard errors.

**Fig. S25 Emission spectrum of MBIC11017 cells grown under white light upon excitation at 440 nm.**


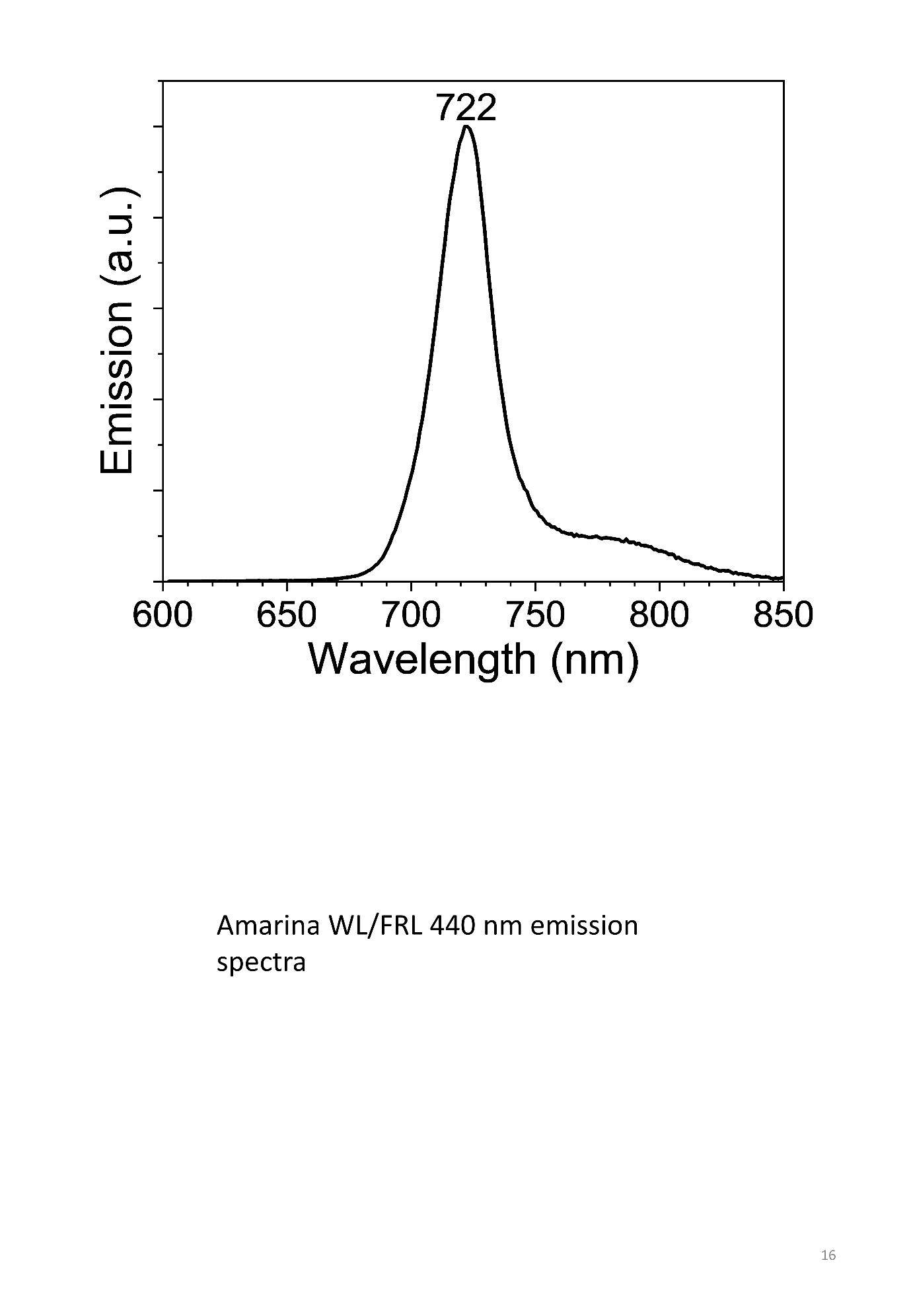


**Fig. S26 Maximum likelihood phylogeny of PsbC, Pcb and IsiA sequences from MBIC11017, *Prochlorococcus marinus* MIT9301, *Prochlorococcus marinus* MIT9313, *Acaryochloris* sp. HICR111A, *Synechocystis* sp. PCC 6803, and *Synechococcus* sp. PCC 7002.** The red box shows the cluster of PsbC sequences, yellow boxes are Pcb proteins from MBIC11017 and Acaryochloris sp. HICR111A and the blue box shows the cluster of IsiA sequences from all cyanobacteria (apart from *Prochlorococcus*). The dark green box shows IsiA sequences from *Prochlorococcus marinus* MIT9301 and *Prochlorococcus marinus* MIT9313, while the light green box shows their Pcb proteins. PsbC sequences were used as an outgroup. The scale represents number of substitutions per site and branch supports are SH-like approximate likelihood ratio tests. See **Methods S4** for details of the tree construction.


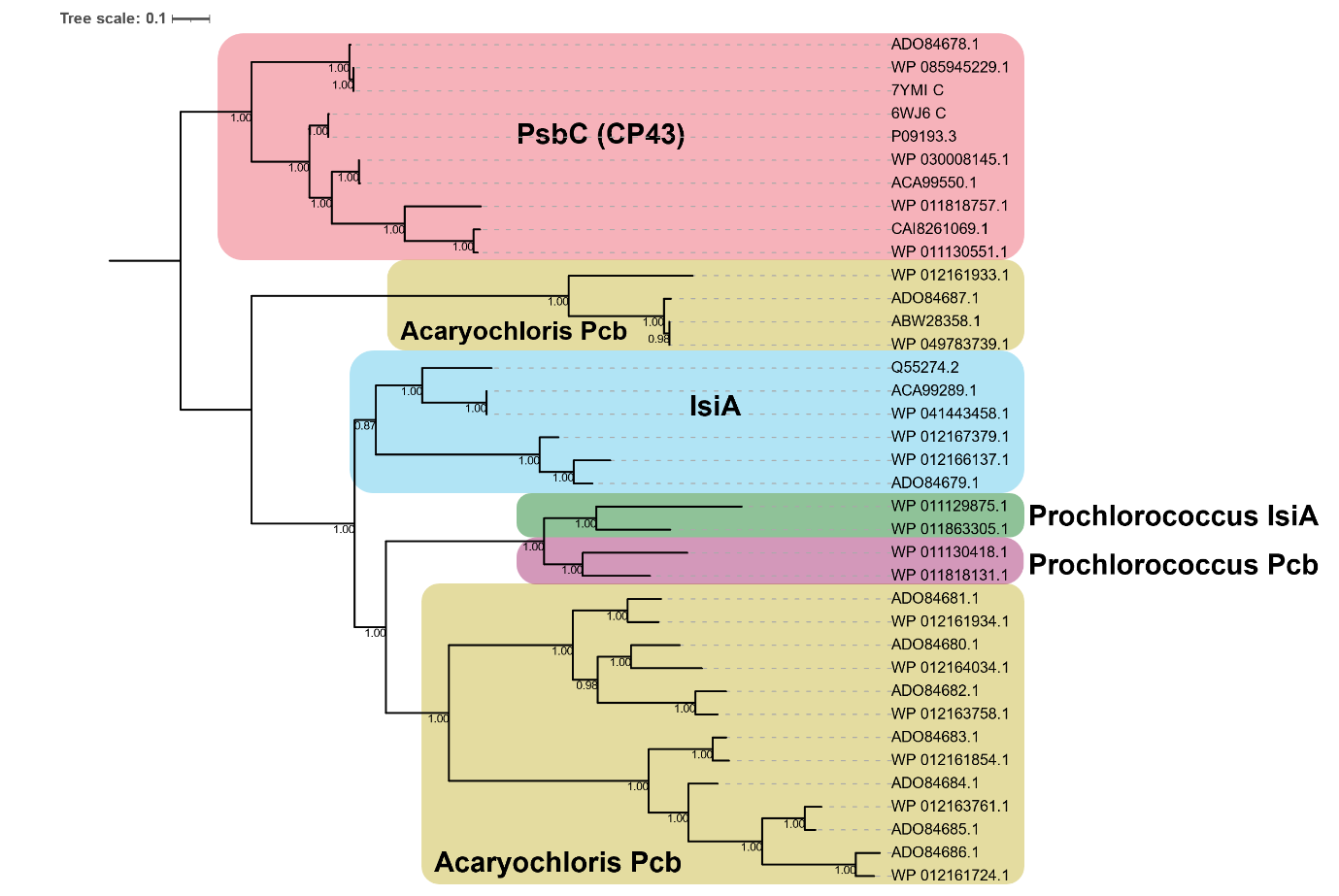


**Fig. S27 Excitation energy trapping dynamics of divinyl-Chl *a*-containing *Prochlorococcus marinus* MIT9301 cells.** Global analysis results for *Prochlorococcus* with PSII in its open state measured using the streak camera setup **(A)** and using the TCSPC setup **(C)**, and the results for the cells measured with PSII in its closed state using the streak camera **(B)** and the TCSPC setup **(D)**. For the TCSPC measurement on *P. marinus* with PSII in its open state the lifetime of the first component was fixed based on the results arising from the streak camera measurements of the cells with PSII in the closed state, since this measurement allows for a better determination of the PSI lifetime due the superior time resolution of the streak and the better separation of lifetimes between PSI and PSII when PSII is its closed state.


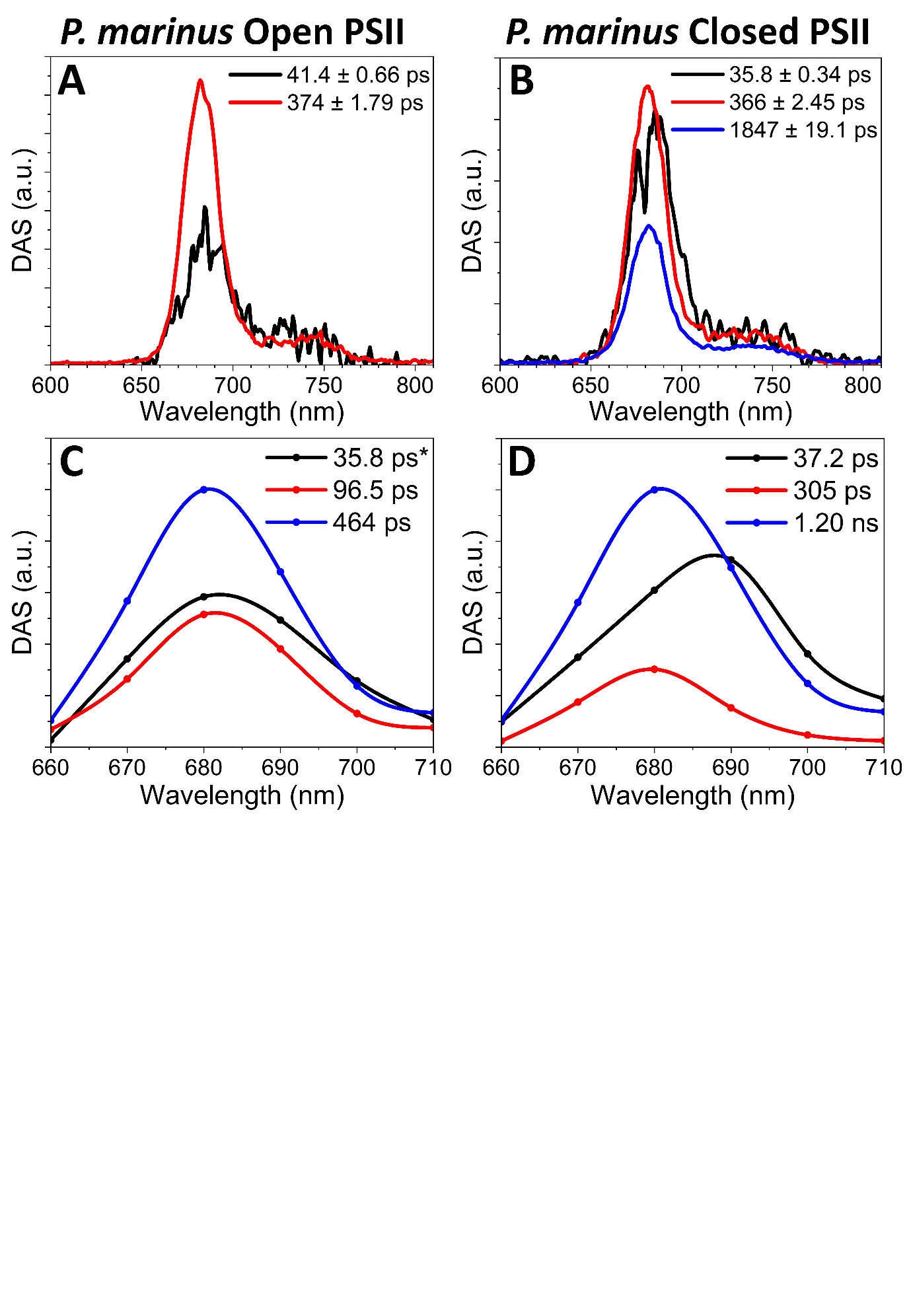


**Table S1** Click here to enter text.Lifetimes and amplitudes for the bi-exponential fits of the energy trapping simulations of MBIC11017 Photosystem II. The simulations shown in **Fig. 6** were performed as described in **Methods S2**. **A_n_** and **τ_n_** denote the amplitude and lifetime, respectively, of each exponential decay component. The average lifetime, **τ_Avg_**, of the trapping dynamics is reported in the last column

|  | **A_1_** | **τ_1_** | **A_2_** | **τ_2_** | **τ_Avg_** |
| --- | --- | --- | --- | --- | --- |
| **Dimeric PSII core** | 0.14 | 0.95 ps | 0.86 | 149.6 ps | 128.7 ps |
| **Dimeric PSII core + 8 Pcb’s** | 0.05 | 1.06 ps | 0.95 | 468.8 ps | 444.3 ps |
| **Tetrameric PSII core + 16 Pcb’s** | 0.05 | 1.06 ps | 0.95 | 466.9 ps | 442.5 ps |

**Methods S1 Streak camera setup description.**

The streak camera setup has been previously described in detail(Hu et al., 2023). In short, a mode-locked Ti:Sa Coherent MIRA oscillator running at 76 MHz was used to seed a Coherent Rega 9050 regenerative amplifier to produce ~80 fs pulses centred around 800 nm at a repetition rate of 250 kHz. These pulses were consequently tuned to either 400 or 580 nm using a Coherent OPA 9400. The FHWM of the pulses was further restricted to 10 nm using interference filters. The excitation pulses were focussed into the cuvette to a spot size of ~100 µm, and the emission was collected at the right angle and focussed into a Chromex 250 IS spectrograph (50 lines/mm, blazed at 600 nm). The output of the spectrograph was focussed onto the input optics of the Hamamatsu C5680 streak camera, which was operated in synchroscan mode and locked to the frequency of the ultrafast oscillator. To achieve acceptable spectral - and time resolutions the spectrograph slit width was set at 100 µm and the streak camera’s photocathode slit width at 40 µm.

For measurements on living cells in open PSII conditions a flow cell cuvette was used to increase the sample refreshment rate within the laser spot. The flow speed was set at ~2.5 ml/s. The sample concentration was 0.3 cm^-1^ at Q_y_ maximum. For the 400 nm excitation measurements a power of 5 µW was used, whereas for the 580 nm measurements a power of 10 µW was used. These powers were at the limit of achieving acceptable signals for the measurements and therefore did not allow for the execution of carefully titrated power studies. To ensure that the large majority of PSII were open during the measurements, the resulting kinetics were compared to TCSPC measurements conducted in parallel, for which instead elaborate power studies were possible (see **Fig. S3**). For the 400 nm excitation measurements of the cells in closed PSII conditions 50 µM of DCMU was added, the samples were pre-illuminated, and measured in a regular 1x1 cm cuvette that contained a magnetic bar revolving at 1200 rpm, at an excitation power of 50 µW.

For the measurements on the isolated PSI complex at room temperature the excitation wavelength was set at 400 nm and the power at 50 µW. The sample was measured in a regular cuvette at a concentration of 0.3 cm^-1^ at Q_y_ maximum and stirred using a stir bar revolving at 1200 rpm. The excitation laser was focussed at the edge of the cuvette to avoid reabsorption. The same sample was also measured in the flow cuvette, which confirmed that its emission dynamics only marginally depend on the redox state of the PSI RC, as is the case for canonical PSI(Wientjes and Croce, 2012). For the measurements on the isolated PSI complex at 77 K the sample was measured in a Pasteur pipette with optical path length of 1 mm, that was submerged in liquid nitrogen. The excitation was set at 400 nm at a power of 150 µW.

**Methods S2 Global and target Analysis.**

The streak camera and TCSPC data Ψ(λ,t) were fitted using a global analysis . In the global analysis the emission data is fitted to a sum of decay-associated spectra DAS(λ) that mono-exponentially decay in parallel with an associated time constant τ, each of them convolved with the instrument response function IRF(t):

$$\Psi\left( \lambda,t \right)=\sum_{i} {DAS}_{i}\left( \lambda\right)\cdot e^{-\frac{t}{\tau_{i}}}\otimes IRF(t)$$

In the case of the streak camera the data was moreover corrected for the dispersion of the emission. The IRF(t) function was modelled as a Gaussian for the streak camera data and measured in the case of the TCSPC data (see TCSPC section). The global analyses for the streak camera data were performed using the pyglotaran python package(van Stokkum et al., 2023, van Stokkum et al., 2004). The fits obtained by the global analysis of the streak camera data can be seen in **Figs. S4** – **S12**. The global analyses for the TCSPC data were performed with the TRFA Data Processor Advanced software(Digris et al., 2014). The fits obtained by the global analysis of the TCSPC data can be seen in **Figs. S13** – **S18**.

A target analysis was performed on the streak camera data of the cells in open PSII conditions excited at 400 and 580 nm, and of the isolated PSI complex, simultaneously, using the pyglotaran Python package(van Stokkum et al., 2023). The kinetic scheme used is shown in **Fig. 5A**. The PSI dataset was described with the sequential model that is indicated with the blue boxes in **Fig. 5A**. The kinetic rates k_PSI1_, k_PSI2_ and k_PSI3_ were fixed to the rates from the corresponding global analysis (see **Fig. 3C**). The weight of the PSI dataset was increased during the fitting such that the arising PSI_1_, PSI_2_ and PSI_3_ SAS matched the corresponding EAS from the global analysis. In this way the PSI dynamics that were determined from the measurements on the isolated system could be used to disentangle the remaining dynamics within the cells. The data on the cells in open PSII conditions for both excitation wavelengths were fitted with the full kinetic scheme of **Fig. 5A**. For the 400 and 580 nm excitation datasets, the initial excitation distribution on PSII and PSI (PSI_1_), and on the PBS (PBS_1_), that are either connected to PSI or PSII, were fitting parameters (see **Fig. 5A**). For the 400 nm dataset the initial excitation distribution over PSI and PSII (J_7_/J_3_) was fixed according to the ratio of the integrated areas of the PSI and PSII DAS arising from the global analysis (**Fig. 2A** & **C**). To reduce the number of free fitting parameters, it was assumed that the 580 nm excitation directly excited PSI and PSII to the same ratio, i.e. J_8_/J_4_ was constrained to the same number as J_7_/J_3_. Similarly, it was assumed that the 400 nm and the 580 nm excitation excited the PBS connected to PSII and those to PSI in the same ratio, and therefore J_1_/J_5_ was constrained to match J_2_/J_6_. To determine the relative J values the areas of the species-associated spectra (SAS) of the PBS_1_, PSI_1_ and PSII compartment had to be constrained(Snellenburg et al., 2013). They were constrained to be equal for PSI_1_ and PSII, and the PBS_1_ compartment was constrained to be 3.67 times higher than that of PSI_1_ and PSII (see **Note S1**). To retrieve SAS of pure PBS species, for the PBS_2_ compartment it was necessary to use the second EAS of the global analysis of the 580 nm excitation dataset as a guidance spectrum and to constrain the PBS_1_ to be zero for wavelengths longer than 725 nm. The natural decay rate for the PBS_2_ compartment, k_PBS4_, was fixed to 0.0005 ns^-1^ in agreement with measurements on the isolated *A. marina* MBIC11017 PBS(Niedzwiedzki et al., 2019). The fits obtained by the target analyses are shown in **Figs. S19** – **20.**

**Methods S3 Excitation energy trapping simulations.**

Excitation energy trapping dynamics within the PSII systems was simulated as described previously (Gradinaru et al., 1998, van Amerongen and van Grondelle, 2000, Croce and van Amerongen, 2020) where the excitation energy transfer (EET) rates between the Chls are calculated based on Förster resonance energy transfer (FRET) theory. In this framework the EET rate from chlorin x to chlorin y, k_xy_ (ps^-1^) is calculated according to:

$$k_{x\to y}=C_{xy}*\frac{\kappa^{2}}{R^{6}}$$

In which R is the centre between the magnesium atoms of the involved Chls (for pheophytin *a* the centre is taken as the geometrical centre of their nitrogen atoms). κ is the orientation factor (see (Gradinaru et al., 1998, van Amerongen and van Grondelle, 2000, Croce and van Amerongen, 2020)). C_xy_ is a constant that encompasses several quantities and in this case depends on the type of Chls involved in the transfer of excitation energy. For transfer between Chls *d* C_xy_ is taken to be 5.59, which is the same as for Chl *a*  Chl *a* EET in (Gradinaru et al., 1998, van Amerongen and van Grondelle, 2000, Croce and van Amerongen, 2020). C_xy_ for EET from a pheophytin *a* to a Chl *d* is taken to be 1.66, whereas for the reverse EET C_xy_ is 0.19, which is analogous to EET between Chl *b* and *a* in the framework of (Gradinaru et al., 1998, van Amerongen and van Grondelle, 2000, Croce and van Amerongen, 2020). In the calculated EET matrix based on this framework an extra compartment is added that represents the PSII RC trap. The EET rate to the trap is set to (1 ps)^-1^ for the 6 RC chlorins and no outgoing EET rates are added for the trap, making it irreversible. For the simulation of the EET dynamics all chlorins in the system are considered to be equally initially excited. The coordinates of the chlorins were extracted from the Protein Data Bank with entry 7YMM(Shen et al., 2023).

**Methods S4 Phylogenetic tree construction.**

Non-redundant protein sequences of Pcb, IsiA or PsbC from MBIC11017, *Prochlorococcus marinus* MIT9301, *Prochlorococcus marinus* MIT9313, *Acaryochloris* sp. HICR111A, *Synechocystis* sp. PCC 6803, and *Synechococcus* sp. PCC 7002 were obtained using the blastp suite on the BLAST online server(Altschul et al., 1990), using the Pcb sequence from Prochlorococcus marinus MIT9313 as a query (NCBI accession no. WP_011129875.1), leaving 37 sequences. These sequences was aligned using the MUSCLE algorithm(Edgar, 2004), with its default parameters, within SeaView(Gouy et al., 2010).

A maximum Likelihood phylogenetic tree was created using the PhyML online server(Guindon et al., 2010). Smart Model Selection(Lefort et al., 2017) using Bayesian Information Criterion was used to determine the most suitable substitution model and parameters. This resulted in the tree being computed the Q.pfam substitution model with invariant sites and four gamma rate categories (Q.pfam+G+I). A starting tree was calculated using the BioNJ method. Branch support was calculated using the SH-like approximate likelihood ratio test. The resulting tree was created using iToL(Letunic and Bork, 2024).

**Notes S1** **The oscillator strength of phycocyanobilin versus that of chlorophyll *d*.**

To determine the EET dynamics of the PBS to the photosystems in a target analysis, it is necessary to have an estimate of the oscillator strength ratio of the PBS phycocyanobilins versus that of the Chls *d*. The molar absorption spectrum of the phycocyanobilins within the *A. marina* MBIC11017 phycocyanin rods has not yet been experimentally determined. As an estimate for the molar absorption spectrum of the *A. marina* MBIC11017 phycocyanin phycocyanobilins we have taken the average molar absorption spectrum of the phycocyanobilins within the C-phycocyanin complex of *Mastigocladus laminosus*(Siebzehnrübl et al., 1987, Sauer et al., 2008). The spectrum was computed by dividing the total molar absorption spectrum of the C-phycocyanin complex by the number of phycocyanobilins that are associated to this complex, which is 3. The area of the resulting absorption spectrum was consequently integrated over the 500-750 nm range. The molar absorption spectrum of Chl *d* within a protein environment has also not yet been determined experimentally, but this has been done for the closely related molecule Chl *a*(Cinque et al., 2000). Density functional theory calculations have predicted the dipole strength of Chl *d* to be roughly 5% higher than that of Chl *a* *in vacuo*(Oviedo and Sanchez, 2011). Moreover, it is shown that the dipole strength of Chls has a linear dependence with the refractive index, and we therefore assume that we can use the same dipole strength scaling factor for the protein environment(Knox and Spring, 2003). In addition, we assume that the red-most band of Chl *a* (and *d*) contains 20% dipole strength from the Q_x_ transition, in accordance with the work of Knox & Spring (2003). After applying these scaling factors, we have integrated the resulting molar absorption spectrum over the 600-750 nm range. By taking into account these data, we could determine an oscillator strength ratio of phycocyanin phycocyanobilin:Chl *d* of 3.67.

**Notes S2** **Comparison of Pcb antenna between *P. marinus* MIT 9301 with *P. marinus* MIT 9313.**

The genomes of both *Prochlorococcus marinus* MIT9301 and *Prochlorococcus marinus* MIT9313 contain one Pcb protein (NCBI accession nos. WP_011863305.1 and WP_011129875.1, respectively). The maximum likelihood tree in **Fig. S26** shows that these two sequences cluster together, forming a distinct clade. It has been shown that this Pcb protein in *P. marinus* MIT 9313 form an antenna system, around the PSII core, forming a PSII-Pcb supercomplex which appears identical in structure to the MBIC11017 PSII-Pcb supercomplex(Bibby et al., 2003). In this work, we measured TRF on *Prochlorococcus marinus* MIT9301 cells (**Fig. S27**). Given that the Pcb sequence from *P. marinus* MIT901 clusters with the Pcb sequence of *P. marinus* MIT9313 (rather than with any other Pcb sequence from e.g. *Acaryochloris* or with IsiA sequences), we assert that the Pcb proteins in MIT9301 also form into a PSII-Pcb supercomplex.

**References**

ALTSCHUL, S. F., GISH, W., MILLER, W., MYERS, E. W. & LIPMAN, D. J. 1990. Basic local alignment search tool. *J Mol Biol,* 215**,** 403-10.

BIBBY, T. S., MARY, I., NIELD, J., PARTENSKY, F. & BARBER, J. 2003. Low-light-adapted Prochlorococcus species possess specific antennae for each photosystem. *Nature,* 424**,** 1051-4.

CINQUE, G., CROCE, R. & BASSI, R. 2000. Absorption spectra of chlorophyll a and b in Lhcb protein environment. *Photosynth Res,* 64**,** 233-42.

CROCE, R. & VAN AMERONGEN, H. 2020. Light harvesting in oxygenic photosynthesis: Structural biology meets spectroscopy. *Science,* 369.

DIGRIS, A. V., NOVIKOV, E. G., SKAKUN, V. V. & APANASOVICH, V. V. 2014. Global analysis of time-resolved fluorescence data. *Methods Mol Biol,* 1076**,** 257-77.

EDGAR, R. C. 2004. MUSCLE: multiple sequence alignment with high accuracy and high throughput. *Nucleic Acids Res,* 32**,** 1792-7.

GOUY, M., GUINDON, S. & GASCUEL, O. 2010. SeaView version 4: A multiplatform graphical user interface for sequence alignment and phylogenetic tree building. *Mol Biol Evol,* 27**,** 221-4.

GRADINARU, C. C., OZDEMIR, S., GULEN, D., VAN STOKKUM, I. H., VAN GRONDELLE, R. & VAN AMERONGEN, H. 1998. The flow of excitation energy in LHCII monomers: implications for the structural model of the major plant antenna. *Biophys J,* 75**,** 3064-77.

GUINDON, S., DUFAYARD, J. F., LEFORT, V., ANISIMOVA, M., HORDIJK, W. & GASCUEL, O. 2010. New algorithms and methods to estimate maximum-likelihood phylogenies: assessing the performance of PhyML 3.0. *Syst Biol,* 59**,** 307-21.

HU, C., ELIAS, E., NAWROCKI, W. J. & CROCE, R. 2023. Drought affects both photosystems in *Arabidopsis thaliana*. *New Phytol,* 240**,** 663-675.

KNOX, R. S. & SPRING, B. Q. 2003. Dipole strengths in the chlorophylls. *Photochem Photobiol,* 77**,** 497-501.

LEFORT, V., LONGUEVILLE, J. E. & GASCUEL, O. 2017. SMS: Smart Model Selection in PhyML. *Mol Biol Evol,* 34**,** 2422-2424.

LETUNIC, I. & BORK, P. 2024. Interactive Tree of Life (iTOL) v6: recent updates to the phylogenetic tree display and annotation tool. *Nucleic Acids Res,* 52**,** W78-W82.

NIEDZWIEDZKI, D. M., BAR-ZVI, S., BLANKENSHIP, R. E. & ADIR, N. 2019. Mapping the excitation energy migration pathways in phycobilisomes from the cyanobacterium Acaryochloris marina. *Biochim Biophys Acta Bioenerg,* 1860**,** 286-296.

OVIEDO, M. B. & SANCHEZ, C. G. 2011. Transition dipole moments of the Q_y_ band in photosynthetic pigments. *J Phys Chem A,* 115**,** 12280-5.

SAUER, K., SCHEER, H. & SAUER, P. 2008. FÖRSTER TRANSFER CALCULATIONS BASED ON CRYSTAL STRUCTURE DATA FROM Agmenellum quadruplicatum C‐PHYCOCYANIN. *Photochemistry and Photobiology,* 46**,** 427-440.

SHEN, L. L., GAO, Y. Z., WANG, W. D., ZHANG, X., SHEN, J. R., WANG, P. Y. & HAN, G. Y. 2023. Structure of a large photosystem II supercomplex from Acaryochloris marina. RCSB Protein Data Bank.

SIEBZEHNRÜBL, S., FISCHER, R. & SCHEER, H. 1987. Chromophore Assignment in C-Phycocyanin from Mastigocladus laminosus. *Zeitschrift für Naturforschung C,* 42**,** 258-262.

SNELLENBURG, J. J., DEKKER, J. P., VAN GRONDELLE, R. & VAN STOKKUM, I. H. 2013. Functional compartmental modeling of the photosystems in the thylakoid membrane at 77 K. *J Phys Chem B,* 117**,** 11363-71.

VAN AMERONGEN, H. & VAN GRONDELLE, R. 2000. Understanding the Energy Transfer Function of LHCII, the Major Light-Harvesting Complex of Green Plants. *The Journal of Physical Chemistry B,* 105**,** 604-617.

VAN STOKKUM, I. H., LARSEN, D. S. & VAN GRONDELLE, R. 2004. Global and target analysis of time-resolved spectra. *Biochim Biophys Acta,* 1657**,** 82-104.

VAN STOKKUM, I. H. M., WEISSENBORN, J., WEIGAND, S. & SNELLENBURG, J. J. 2023. Pyglotaran: a lego-like Python framework for global and target analysis of time-resolved spectra. *Photochem Photobiol Sci,* 22**,** 2413-2431.

WIENTJES, E. & CROCE, R. 2012. PMS: photosystem I electron donor or fluorescence quencher. *Photosynth Res,* 111**,** 185-91.
